# Diet-induced metabolic and faecal microbiome responses in pet dogs fed a minimally processed versus extruded kibble diet

**DOI:** 10.3389/fvets.2026.1734572

**Published:** 2026-04-10

**Authors:** Louise Campbell, Mary Thompson, Matthew Muir, David Raubenheimer, Andrew Holmes

**Affiliations:** 1 Charles Perkins Centre, University of Sydney, Sydney, NSW, Australia; 2 School of Life and Environmental Sciences, University of Sydney, Sydney, NSW, Australia; 3 Faculty of Science, Sydney School of Veterinary Science, The University of Sydney, Sydney, NSW, Australia; 4 Lyka Pet Food, Sydney, NSW, Australia

**Keywords:** blood glucose response, canine nutrition, companion animal health, extruded kibble, food processing, gut hormone, gut microbiome, minimally processed diet

## Abstract

**Introduction:**

The extent to which food is processed influences gut functions, such as digestion, nutrient uptake, and microbiome response, with the potential to impact immuno-metabolic health. Despite known associations between ultra-processed food (UPF) intake and chronic diseases in humans, mechanistic links explaining inferred health risks remain unclear. Health risks associated with chronic disease are also a concern for companion animals such as dogs, where nutritionally complete dry processed foods constitute a major component of contemporary pet dog diets. A challenge is that nutrition is complex and even diets based on nutritionally complete processed foods typically vary in multiple dimensions.

**Methods:**

Here, we use a randomised, cross-over design to compare short-term effects of two commercially available nutritionally complete canine diets—an extruded kibble diet (EKD) and a mildly cooked, minimally processed diet (MPD). We assigned 24 healthy dogs to one of two groups: all dogs remained on their at-home diet for 1 week, before transitioning to (1) MPD or (2) EKD for 2 weeks each. One dog was removed from the study due to health complications, and the remaining 23 dogs completed both dietary treatment periods. Glycaemic, hormonal, and gut microbiome responses were captured from pre- and post-prandial blood samples and time series of 12 faecal samples from each dog.

**Results:**

The experimental diets resulted in distinct physiological and gut microbiome responses. MPD was associated with improved faecal consistency (FCS 2.24 ± 0.67, *p* = 0.005), lower pre- and post-prandial gastric inhibitory polypeptide (GIP, p < 0.001) and peptide YY (PYY, *p* < 0.05), and reduced post-prandial glycaemic response compared to EKD (AUC, *p* = 0.009). Diet was the strongest predictor of microbial response despite between-dog differences, with higher alpha diversity associated with MPD and greater within-individual community turnover following transition to MPD in both treatment groups (Group 1, *p* < 0.001; Group 2, *p* < 0.05).

**Conclusion:**

These data show the feasibility of testing diet formulations in pet dogs and understanding the role of host–microbiome interactions in responses to food. The demonstration of distinct outcomes highlights the need for further studies on long-term feeding to better understand mechanisms and implications for health in community pets.

## Introduction

Nutrition-related chronic diseases (NRCDs), such as obesity, diabetes mellitus, and chronic inflammatory conditions, are an increasing epidemic in modern society for humans and our companion animals (1–6). In animal models with experimentally defined diets, it is well established that diet is sufficient to trigger such diseases via mechanisms that are not simply related to nutrition and metabolism but also include the immune and nervous systems (7–9). Each of these systems is influenced by gut microbial activity—changes in which are commonly associated with NRCDs—and the gut microbiome is now recognised as an important interface between the host and ingested foods (10–13). Interaction between diet and the microbiome influences numerous physiological processes such as gut functions related to stool formation, bile resorption, epithelial integrity and intestinal motility; digestive processes that underpin efficiency of energy and nutrient harvesting from food (14, 15); and production of small molecules with bioactive effects such as inputs for gut–brain signalling in homeostatic regulation, or adjustment of immune tone (16, 17). Consequently, understanding how ingested foods and the gut microbiome interact is now recognised as an additional factor when assessing the quality of diets for sustaining health.

This issue is especially important for the food industry, wherever a food product is intended to be a major component of the diet. Processed foods can reconfigure gut microbiome substrates with downstream effects on physiology and eating behaviour (18). In humans, there is strong evidence of a correlation between the proportion of processed foods in the diet and NRCD prevalence (19–22). The epidemiological link between ultra-processed foods (UPFs) and chronic disease in humans has coincided with increased awareness of chronic conditions such as obesity and diabetes in pet dogs (23–26), although direct associations between food processing level and chronic disease have not been established in this species. Pets may be especially exposed since processed foods comprise the dominant component of the diets of many domestic dogs and cats (2). Evidence that dietary exposures across early and later life influence long-term health outcomes in dogs further supports the importance of understanding how different dietary formulations interact with host physiology and the gut microbiome (1, 27, 28). This growing interest highlights the need for studies examining how commercially available diets influence host responses.

A significant body of human research has focused on macronutrient distribution and quality in mass-produced, long shelf-life products (29–31). Such products are relatively inexpensive, highly convenient, and dominate the market. Similarly, “extruded kibble” dry foods comprise a large proportion of most dogs’ daily diet. More recently, minimally processed dog foods—which may be raw or gently cooked (~80 °C)—have entered the pet food market. Both foods are formulated to be nutritionally complete; however, the kibbles are structurally and chemically distinct from minimally processed cooked diets due to extensive industrial processing, including high-temperature extrusion, maceration, and the use of synthetic preservatives and palatants (32). It has been proposed that associations between UPFs and the onset of chronic diseases may arise from factors such as reduced digestibility caused by extrusion and thermal processing effects (32–34), altered nutrient absorption due to changes in food structure and matrix (35, 36), or bioactive effects of food additives and processing-derived compounds such as advanced glycation end products (20, 22, 37, 38). Although minimally processed pet foods are marketed as more “natural” compared to traditional extruded kibble diets (39–42), it is currently unclear to what extent extruded kibble and minimally processed foods differ in their effects on pet health or interaction with the microbiome.

All animals and their microbiomes have evolved mechanisms to adaptively respond to foods encountered in the natural environment over time (43). An example of such long-term co-evolution is the emergence of distinct gut microbial community types—termed “enterotypes” by Arumugam et al. (44)—whose prevalence differs across human populations at different stages of food system modernisation. Understanding how such changes affect health is essential (20, 21). A key question is distinguishing whether risks are associated with specific UPF products (item-level risk) or with overall dietary patterns (diet-level risk) that alter feeding behaviour, immuno-metabolic homeostasis, and bioactive properties of components. This distinction is particularly relevant when assessing foods formulated to be nutritionally complete, such as kibble diets, which nonetheless share defining characteristics with UPFs.

To better understand how diets that are considered nutritionally complete, yet differ in ingredient composition and manufacturing approach, may influence health outcomes over time, we conducted a cross-over feeding study comparing a minimally processed diet (MPD) and an extruded kibble diet (EKD). We aimed to determine whether (1) metabolic signalling (appetite-control hormones and cytokines) differed between diets; (2) the gut microbiome showed diet-associated compositional or functional changes; and (3) individual variation in host responses was linked to the dogs’ microbiome state. We designed the study such that dogs remained in their normal at-home environment and chose commercially available pet foods to ensure real-world applicability of our study. A cross-over design controlled for individual variability, with 24 healthy pet dogs randomly assigned to one of two diet sequences: all dogs remained on their at-home diet for 1 week, before being gradually transitioned to (A) MPD or (B) EKD for 2 weeks as their first experimental diet. Dogs were then transitioned to their second experimental diet.

We monitored broad biometrics (bodyweight and faecal consistency), physiological outcomes (glycaemic, hormonal, and cytokine measures), and gut microbiome (faecal) responses. Fasting and post-prandial blood measurements were used to capture responses across multiple temporal scales, with fasting samples obtained after each 2-week feeding period reflecting a diet-adapted physiological state and post-prandial samples capturing acute responses to meal composition. In parallel, repeated faecal sampling enabled separation of short-term variability from diet-associated shifts in the stable gut microbial community. We hypothesised that microbiome composition and host metabolic profiles would differ between MPD and EKD, and tested for the possibility that between-dog differences in microbiome state were predictive of response.

## Materials and methods

### Ethical approval and study design

The study was submitted to and approved by the Animal Ethics committee of the University of Sydney (2023/AE02299).

We recruited 24 privately owned dogs to the study after pre-screen surveys and a preliminary clinic visit at the University Veterinary Teaching Hospital Sydney (UVTHS) for body condition scoring and behavioural assessment. Each dog was weighed and assigned a body condition score (BCS) according to Laflamme (45). Dogs were eligible if they showed no undue distress under clinic handling conditions, were between 2 and 10 years old, weighed over 10 kg, BCS in the range of 4–7, and no health issues were detected. Specific exclusions included no chronic health conditions, no history of chronic or recurrent gastrointestinal issues, no known food intolerances or allergies, no antibiotic use within 3 months prior to recruitment, and no current medication use or prescribed diet. A further assessment of study participation was made by our veterinarian when dogs were reviewed during their baseline visit. Three dogs were deemed unsuitable for blood collection owing to apparent anxiety but retained in the study for microbiome outcomes. Dogs were to be removed if they experienced significant difficulty in completing their meals during either intervention or if dogs responded adversely to the new diets (in consultation with our veterinarian). A target sample size of 24 dogs was selected based on precedent from comparable dietary intervention studies, with a crossover design used to maximise within-dog power in this pilot study.

Recruited dogs were arbitrarily assigned to one of two experimental diet-sequence groups:

*Group 1*: fed MPD first, followed by EKD.

*Group 2*: fed the diets in reverse order, EKD followed by MPD.

### Baseline metrics

Owners were asked to collect three faecal samples over the course of 1 week, while dogs remained on their at-home diet (Supplementary Figure 1). Where possible, owners attempted to collect all faecal samples throughout the study at a consistent time across collection days, with a preference for the morning. Owners completed a FCS (faecal consistency score, Nestlé Purina, Vevey, Switzerland) for each collection, as well as a longer survey about their dog’s dietary habits. At the time of recruitment, no dogs were being fed either of the experimental diets. As expected, kibble foods comprised part or all of the baseline diet for most dogs (22/24) (Supplementary Table 2A).

At their first clinic visit, a fasting blood sample was taken (minimum 12-h fast) from all suitable dogs before they were fed their usual, at-home breakfast (provided by the owner). Dogs were given 15 min to eat their meal, and the proportion of supplied food eaten was recorded to the nearest quartile. Following this, a blood glucose timeseries was measured using an AlphaTrak2 glucometer at *T* = 0, 15, 30, 45, 75, 105, 135, 165, 195, and 240 min. A second (postprandial) blood draw was taken at *T* = 4 h (240 min).

### Experimental diet period

Dogs were gradually transitioned onto the first of two experimental diets over the course of 1 week (according to group assignment). Transition involved feeding a mixture of 25% of the new experimental diet with 75% of their baseline diet for 3 days, followed by a 50:50 mixture for 2 days, and a 75:25 mixture for 2 days. They were then exclusively fed the experimental diet for 2 more weeks before returning to the veterinary clinic for a second set of blood draws and glucose measurements. Owners were asked to minimise treats to ensure the experimental diet comprised their dog’s main diet, and treats accounted for less than 10% of their dog’s daily energy intake. Follow-up veterinary clinic visits were as close to the 14th day of the experimental diet as possible for the owners/veterinary clinic (with 9 days of experimental diet feeding accepted as the minimum period). All but four clinic visits were conducted on the 12th–14th day (i.e., 43/47, with one dog withdrawing before their final visit).

While the dog was being fed their first experimental diet, owners were asked to collect three faecal samples on three specified days (as outlined in Supplementary Figure 1) and complete corresponding collection surveys as well as two separate diet surveys for their dogs. Diet surveys were primarily used to monitor diet adherence and signs of adverse reaction. Following their second visit (or after completion of the 14 experimental diet days, if the visit occurred before this), dogs were gradually transitioned onto their second experimental diet following the same procedure as for the first, this time eating a graded mixture of their first and second experimental diets. Owners were asked to collect an additional three faecal samples during this transition period. Feeding, sample collection, and surveys were then repeated as for their first experimental diet. Dogs completed the study following a third visit to the veterinary clinic.

### General characteristics of the study population

A total of 24 healthy dogs (11 male; 13 female) of 18 different breeds (including mixed breeds, counted separately) with an age range of 2–9 years and bodyweight range of 11–43 kg were recruited (Supplementary Tables 2A,B). At baseline, average BCS was 5.33 ± 0.86, with 13 in the ideal range (4 or 5) and eight overweight (6 or 7). BCS was not recorded for the three dogs previously noted as unsuitable for blood collection due to anxiety. One dog in Group 2 was removed from the study (both blood and stool sampling) after 4 weeks when they demonstrated intolerance to the second diet treatment (MPD). Data collected before withdrawal were retained for analyses where applicable, with missing observations accommodated by mixed-effects modelling.

### Diet formulations and portion calculations

MPD (Lyka Chicken Bowl) and EKD [Hill’s Science Diet Healthy Mobility (Adult)] are commercially available diets. EKD is a dry, extruded kibble diet, whereas MPD is produced by mincing and cooking ingredients at a maximum temperature of 90 °C in steam-jacketed kettles, followed by freezing at −18 °C before distribution. We therefore describe MPD as a “minimally processed” commercial diet defined as undergoing a single primary thermal step before cold-chain preservation (46). Full nutrient composition as provided by the manufacturers is in Supplementary Tables 1A–C, and both proximate and manufacturer-provided macronutrient energy distributions are in Table 1. Proximate composition of MPD was determined by independent third-party analysis and reported on an as-fed basis (g/100 g). Two proximate analyses were available for the trial period; mean values were calculated for moisture, ash, crude protein, and total fat. One crude fibre value was reported below the laboratory reporting limit (<1.0 g/100 g) and was imputed using midpoint substitution. Total dietary fibre (TDF) was measured independently using an enzymatic-gravimetric method (AOAC 991.43) and converted to a dry matter basis using measured moisture content. Batch-matched proximate analysis could not be performed for EKD due to reformulation; therefore, TDF was not reported for EKD.

**Table 1:** Macronutrient and fibre composition of Lyka Chicken Bowl and Hill’s Science Diet Healthy Mobility (Adult), using third-party and manufacturer-reported analyses, respectively.

Nutrient	As-fed (g/100 g) Lyka Chicken Bowl^a^	Dry matter (%) Lyka Chicken Bowl	Dry matter (%) Hill’s Healthy Mobility^b^	% of calories Lyka Chicken Bowl^c^	% of calories Hill’s Healthy Mobility^c^
Moisture	74.4	—	—	—	—
Energy	1,023 kcal/kg	3,994 kcal/kg	3,617 kcal/kg	—	—
Protein	15.2	59.4	22.4	52.0	19.9
Fat	4.35	17.0	14.7	36.2	31.7
Carbohydrate/NFE	3.45	13.5	54.2	11.8	48.2
Crude fibre (CF)	0.8	3.1	2.4	—	—
Total dietary fibre (TDF)	1.4 ± 0.1	5.5 ± 0.4	Data not available	—	—

a Lyka Chicken Bowl values are based on two independent third-party proximate analyses conducted during the trial period. Mean moisture content corresponded to a dry matter content of 25.6%. One crude fibre value reported as below the laboratory reporting limit (“<1.0 g/100 g”) was treated as 0.5 g/100 g (midpoint substitution) for the calculation of the mean crude fibre concentration. Total dietary fibre (TDF) was measured independently using an enzymatic-gravimetric method (AOAC 991.43) and is reported as mean ± SEM.

b Hill’s Healthy Mobility dry matter composition and metabolisable energy were obtained from manufacturer-reported data for the formulation used during the study period. Total dietary fibre (TDF) data were not available for this formulation.

c Macronutrient energy distribution (% of calories) for both diets was calculated from dry matter macronutrient composition using modified Atwater factors (protein 3.5 kcal/g, fat 8.5 kcal/g, carbohydrate/NFE 3.5 kcal/g). Fibre was not assigned a caloric value in these calculations.

For contextual comparison, the macronutrient energy profile of MPD was plotted relative to published datasets of commercially available dry kibbles (47) and wet canned dog foods (48) using a right-angled mixture triangle (49) (Supplementary Figure 2). Percent metabolisable energy (%ME) from protein and fat is derived using reported metabolisable energy values (modified Atwater factors where applicable), with the third macronutrient calculated as 100 − (protein %ME + fat/carbohydrate %ME).

Portion guidelines were aimed to ensure dogs maintained their bodyweight throughout the study. The manufacturers’ approach to portion recommendation differed slightly, with the manufacturer of MPD using a proprietary model to personalise an amount for each dog and the EKD manufacturer using a generic guide based on bodyweight only (as with most dry foods). For MPD, the manufacturer provided food sachets in daily portions determined according to their guidelines for meeting metabolisable energy requirements. For EKD, owners were instructed to feed their dogs a weighed portion of food, calculated using a standard metabolisable energy equation based on resting energy requirement (RER), multiplied by a life stage factor, consistent with AAHA Nutrition and Weight Management Guidelines (50) for adult, neutered dogs:


$$\mathrm{ME}=\mathrm{RER}\;x\;1.5,\text{where}\;(\mathrm{RER}={\text{bodyweight}}^{0.75}\times 70)$$

### Blood sample processing

Blood collection and processing were performed using standardised laboratory procedures routinely used in our laboratory. A total of 2 × 4 mL blood samples were taken from each dog per clinic visit. A total of 3 mL of each sample was kept on ice in uncoated serum tubes, while the remaining 1 mL was aliquoted into a lithium-heparin tube. Blood stabilisers were added to the serum tubes to minimise protease degradation. Immediately following collection of the *T* = 4 h blood sample, all serum samples were incubated at room temperature for 30 min and centrifuged at 1,000 *g* for 10 min at room temperature, while the plasma samples were centrifuged at 1,000 *g* for 10 min at 4 °C. All samples were aliquoted and stored at −80 °C until required for analysis.

### Gut appetite hormone multiplex assay and analysis

Canine gut and appetite hormones were quantified from fasting (*T* = 0 h) and 4-h post-prandial (*T* = 4 h) serum samples using a MILLIPLEX® Canine Gut Hormone Magnetic Bead Panel on a Luminex MAGPIX system, as per the manufacturer’s protocol. Analytes included total amylin, active ghrelin, glucagon-like peptide-1 (GLP-1), gastric inhibitory polypeptide (GIP), pancreatic polypeptide (PP), peptide YY (PYY), insulin, glucagon, and leptin. Hormone data were analysed in GraphPad Prism using mixed-effects models (REML), with timepoint and diet included as fixed effects and dog included as a repeated-measures (random) effect. Multiple comparisons were adjusted using Tukey’s test.

### Cytokine, chemokine, and growth factor multiplex assay and analysis

Canine cytokines, chemokines, and growth factor were quantified for post-prandial serum samples (*T* = 4 h) using a ProcartaPlex™ Canine Cytokine/Chemokine/Growth Factor Panel 1, 9plex on a Luminex MAGPIX system, as per the manufacturer’s protocol. Analytes included were interferon-gamma (IFN-γ), interleukin-2 (IL-2), interleukin-6 (IL-6), interleukin-8 (IL-8), interleukin-10 (IL-10), interleukin-12/23p40 (IL-12/23p40), monocyte chemoattractant protein-1 (MCP-1), tumour necrosis factor-alpha (TNF-α), and vascular endothelial growth factor-A (VEGF-A). Cytokine and growth factor concentrations were analysed in GraphPad Prism using mixed-effects models (REML), with diet included as a fixed effect and dog included as a random effect, and Tukey’s test was used to adjust for multiple comparisons where applicable. Several analytes were frequently below the assay’s limit of detection or quantification, and only analytes with >50% of samples above the detection threshold were included in statistical analyses.

### Faecal sample storage and DNA extraction

All faecal samples were collected in faecal collection containers and stored as soon as possible in the owner’s freezer (approximately −20 °C) until they could be returned to the researchers (1–2 weeks). These samples were then stored in a −30 °C freezer before DNA extraction in accordance with standardised procedures routinely used in our laboratory. DNA extraction was performed using a SPINeasy® DNA Pro Kit for Feces (MP Biomedicals) according to the manufacturer’s protocol, and DNA was stored in a −80 °C freezer until sent for sequencing. DNA concentration and integrity were assessed for all samples before sequencing using fluorometric quantification (Qubit), PCR amplification, and gel electrophoresis.

### 16S amplicon sequencing and analysis

Faecal bacterial communities were profiled using the 16S rRNA V3-V4 region. Library preparation and sequencing were conducted by the Ramaciotti Centre for Genomics (UNSW, Sydney, Australia) using an Illumina MiSeq2. Raw paired-end sequences were cleaned of adapter/primer sequences using Cutadapt (51) before being processed and filtered using the DADA2 (52) (1.8.0) pipeline in R (4.4.1). Samples failing quality control or filtering thresholds during DADA2 processing were excluded from downstream analyses. Taxonomy was assigned using the SILVA (53) v138.1 database. A phylogenetic tree was constructed using multiple sequence alignment with ClustalW (54) and a generalised time-reversible model in phangorn (55) v2.12.1. The final ASV table, taxonomy table, and phylogenetic tree were integrated into a phyloseq (56) object for downstream analyses.

Samples were rarefied to an even sequencing depth (Supplementary Figure 3). Alpha diversity (Observed richness, Shannon, and Inverse Simpson) was calculated at ASV, Genus, Family, and Phylum levels. Kruskal–Wallis tests with Dunn’s *post-hoc* comparisons (Benjamini–Hochberg correction) assessed statistical differences, and ggplot2 (57) was used for visualisation. Beta diversity was assessed using Bray–Curtis, unweighted UniFrac, and weighted UniFrac. A rooted phylogenetic tree was used to calculate these distances, with data CLR-transformed. Principal Coordinate Analysis (PCoA) was performed to visualise the samples in ordination plots. PERMANOVA using the adonis2() function from the vegan (58) v2.7-0 package tested for statistical significance between groups. db-RDA was performed using the capscale() function from vegan, with diet as the explanatory variable and data Hellinger-transformed. Significance was assessed using permutation tests (999 permutations) via the anova() function. LEfSe (59) and ALDEx2 (60) were used for differential abundance analysis of taxa at the ASV and genus level. LEfSe was run using the default workflow combining a Kruskal–Wallis test with linear discriminant analysis (LDA) to identify differentially abundant features; no additional external false discovery rate (FDR) correction was applied. LEfSe analyses were performed with diet as the primary class, pooling diet sequence groups to maximise statistical power for detecting diet-associated taxa. ALDEx2 was applied as a complementary differential abundance approach incorporating compositional data analysis and explicit FDR control.

To assess whether samples formed discrete groups based on overall ASV- or Family-level community composition, we performed exploratory clustering analyses using partitioning around medoids pam() function from the cluster (61) package. Before clustering, ASV and family abundance tables were centre-log-ratio (CLR) transformed to mitigate compositional effects. Pairwise sample dissimilarities were calculated using Bray–Curtis distances. Clustering solutions across a range of cluster numbers (*k* = 2–10) were evaluated using the Calinski–Harabasz index, and, drawing from this, clustering was run for *k* = 2 and *k* = 3. Results were visualised using the fviz_cluster() function. Multipatt analysis was performed to test for significant associations between clusters and ASVs and families by permutation testing (999 permutations) using the multipatt() function from the indicspecies (62) package.

Gene frequencies and potential metabolic traits were inferred from our ASV data using PICRUSt2 (63) and MetaCyc (64) pathways, and Kegg Orthologs (65) were averaged and CLR-transformed across each diet. Differential abundances of pathways were statistically tested using ALDEx2. GitHub Copilot v1.168.0 and ChatGPT (OpenAI GPT-4 model) were used to assist with script development in RStudio and Bash (Unix shell environment).

### Statistical analysis

Statistical analyses were conducted using GraphPad Prism (for blood-based outcomes) and R (v4.4.1) for microbiome analyses. All tests were two-sided, and statistical significance was defined as *p* < 0.05. Where multiple pairwise comparisons were performed within an outcome, *p*-values were adjusted using Tukey’s multiple comparisons test or Benjamini–Hochberg false discovery rate control, as appropriate. Mixed-effects modelling was used for blood-based outcomes to account for repeated measures and inter-individual variability, allowing inclusion of all available observations in the presence of missing data. For outcomes analysed using paired tests rather than mixed-effects models (e.g., bodyweight), only dogs with complete paired measurements were included. Microbiome community-level differences were assessed using distance-based, permutation methods. No formal statistical outlier detection or exclusion criteria were applied.

## Results

### Biometric and physiological outcomes across the diet sequence

The mean bodyweight for all dogs in the study did not change significantly between their starting (baseline) and final timepoint (mean increase +1.3%; *p* = 0.16) (Supplementary Table 3), although individual dogs did show minor weight fluctuations associated with diet transitions. Of note, neither Group 1 nor Group 2 dogs experienced a significant change in bodyweight between baseline and their first dietary treatment (−1.3%, *p* = 0.14; +0.4%, *p* = 0.56, respectively). However, Group 1 dogs showed a significant increase in bodyweight when transitioned from their first to their second experimental diet (MPD – EKD; +3.1%, *p* = 0.011), whereas no significant change was observed in Group 2 (EKD – MPD; −0.8%, *p* = 0.58). We note that this Group 1 transition was also associated with greater microbiome impact (see below). Owner-reported faecal consistency scores on MPD were closest to an ideal score of 2 (2.24 ± 0.67) and were significantly lower compared to both EKD and baseline (BL) (2.87 ± 1.29 and 2.69 ± 0.96, respectively; *p* < 0.005) (Supplementary Table 4).

**Figure 1:**
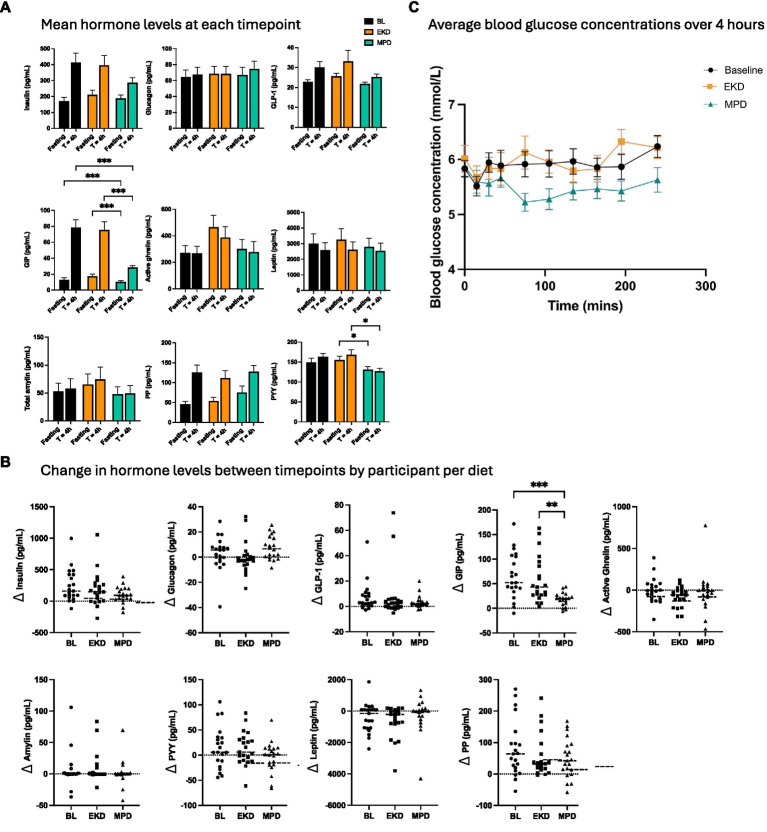
Pre- and post-prandial hormone and glucose responses after a meal. **(A)** Average concentrations of gut- and appetite-related hormones from fasting and 4 h post-prandial serum samples, according to diet (BL, EKD, and MPD). **(B)** Difference in hormone levels between timepoints for all participants (*n* = 21 dogs; one missing observation on MPD). **(C)** Averaged post-prandial blood glucose curves across all participants who underwent blood glucose monitoring (*n* = 21 dogs; one missing observation on MPD) from *T* = 0 to 4 h. Data were statistically tested using a mixed effects model and multiple comparisons tests. Error bars represent ±SEM (^*^p < 0.05; ^**^p < 0.01; ^***^p < 0.001).

Nine hormones were measured in pre- and post-prandial samples at the end of each feeding period. Mean GIP levels were significantly lower in dogs on MPD than EKD and BL at both *T* = 0 and *T* = 4 h (Figure 1A) (*p* < 0.001). Mean PYY levels were also significantly lower on MPD than EKD at *T* = 0 and *T* = 4 h (*p* < 0.05). No statistically significant differences in mean concentrations were seen for other hormones. However, consistent directional differences were observed following the 2-week dietary interventions, suggesting longer periods on diet or larger sample sizes may be worth investigation (Supplementary Table 5). Fasting active ghrelin concentrations were higher on EKD than MPD in 13 of 20 dogs, with a median within-dog difference of ~60%, with a smaller median separation post-prandially (~22%). Fasting leptin concentrations were also higher on EKD in 15 of 20 dogs, with a median difference of ~30%. In contrast, hormones primarily involved in post-prandial nutrient signalling showed larger separation between diets after feeding, e.g., post-prandial insulin concentrations were higher on EKD in 16 of 20 dogs (median difference ~30%), despite modest fasting differences.

We also assessed the magnitude of hormone change after feeding in participants (Figure 1B). These data emphasise the consistent attenuating effect of MPD on GIP response compared to EKD across all dogs, relative to greater individual variability in response for other tested hormones (e.g., ΔPYY concentrations had a similar range across all three diets). When data were split by treatment group, no overt effect of diet sequence was seen, although some hormone responses differed. For example, total amylin was numerically highest on EKD in Group 1, and PYY increased post-prandially in Group 1 but decreased in Group 2 (Supplementary Figure 5). No consistent relationship was observed between meal incompletion, bodyweight or age, and outlying hormone response patterns (Supplementary Table 6).

Postprandial glycaemic response showed group-level differences between diets. For both treatment order groups, the average AUC_total_ was significantly lower when the dogs were fed MPD compared to EKD (*p =* 0.009). Peak blood glucose concentration (*p* = 0.007) and times to peak (*p* = 0.005) were also significantly lower on MPD than EKD. Despite these overall effects, substantial inter-individual variability in response was observed with blood glucose timeseries for each individual, highlighting that, although a lower glycaemic response when on the MPD diet was the most common observation, this was not universal. For example, dogs P01 and P32 had a lower response on EKD (full data in Supplementary Figure 6, Table 7).

Of the cytokines and growth factors measured, only MCP-1, IL-8, IL-12/IL-23p40, and VEGF-A were detectable in more than 50% of samples (Supplementary Figure 4A). VEGF-A concentrations were nominally higher on MPD than EKD (*p* < 0.05) (Supplementary Figure 4B); however, this finding was based on a limited number of detectable samples and was not interpreted further.

Overall, these physiological measures indicated no overt adverse effects of either experimental diet, modest improvements in faecal consistency and glycaemic control on MPD, and substantial inter-individual variability in response.

### Gut microbiome alpha diversity metrics indicate diet and treatment-dependent shifts in the partitioning of resources

**Figure 2:**
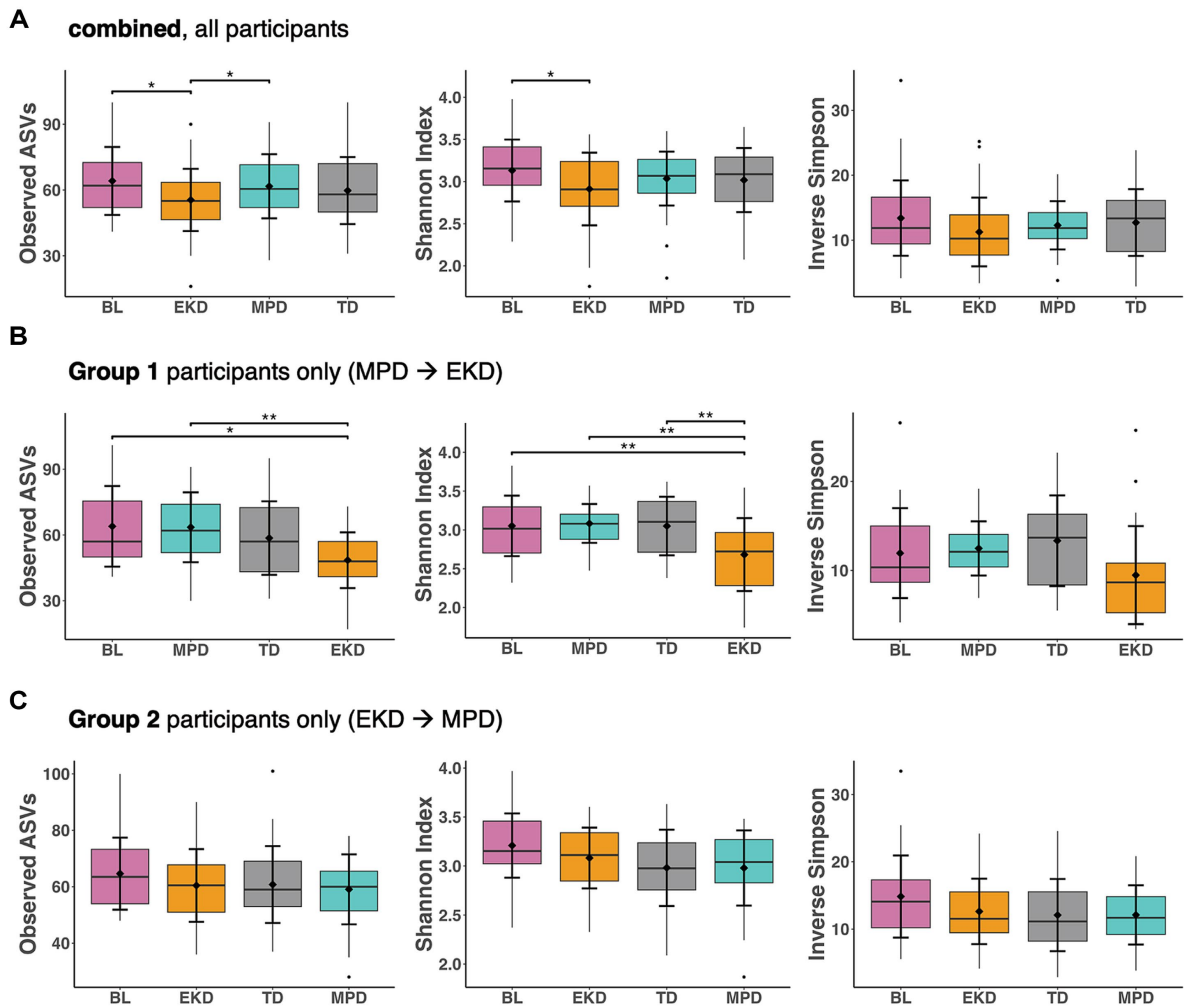
Alpha diversity metrics of observed species, Shannon’s Index, and Inverse Simpson’s Index for **(A)** all participants (*n* = 24 dogs, multiple samples per dog); **(B)** Group 1 participants (BL—MPD—TD—EKD sequence); and **(C)** Group 2 participants (BL—EKD—TD—MPD sequence), using Kruskal–Wallis multiple comparison test with *p*-values adjusted using Benjamini–Hochberg method (^*^p < 0.05, ^**^p < 0.01, ^***^p < 0.001).

To characterise the range of complexity in microbial communities, we examined multiple alpha diversity metrics. Across all participants, alpha diversity metrics showed greater median values on MPD than EKD (Figure 2A). Observed ASV richness was significantly greater on MPD than EKD (*p* < 0.05), while Shannon’s index was significantly lower on EKD compared to baseline (*p* < 0.05). When analyses for each diet sequence group were examined separately, differences in observed ASVs and Shannon’s index were more pronounced in Group 1 and were not statistically significant in Group 2 (Figures 2B,C). At baseline, most dogs (22/24) were consuming extruded kibble as a substantial component of their habitual at-home diet, which may have contributed to BL microbiome states more similar to EKD than MPD. In Group 1 individuals, observed ASVs on EKD were significantly lower than both BL and MPD (*p* < 0.01; *p* < 0.001), and Shannon’s index was significantly lower than BL, MPD, and TD (transition period between experimental diets 1 and 2) (*p* < 0.001) (Figure 2B). Together, these findings suggest that resource partitioning of ecological space in the gut communities differed between the diet regimens (with EKD supporting lower diversity than MPD or the range of BL diets) and that the diet sequence may also influence the community response to disturbance.

### Importance of individuality and microbial state in shaping response

**Figure 3:**
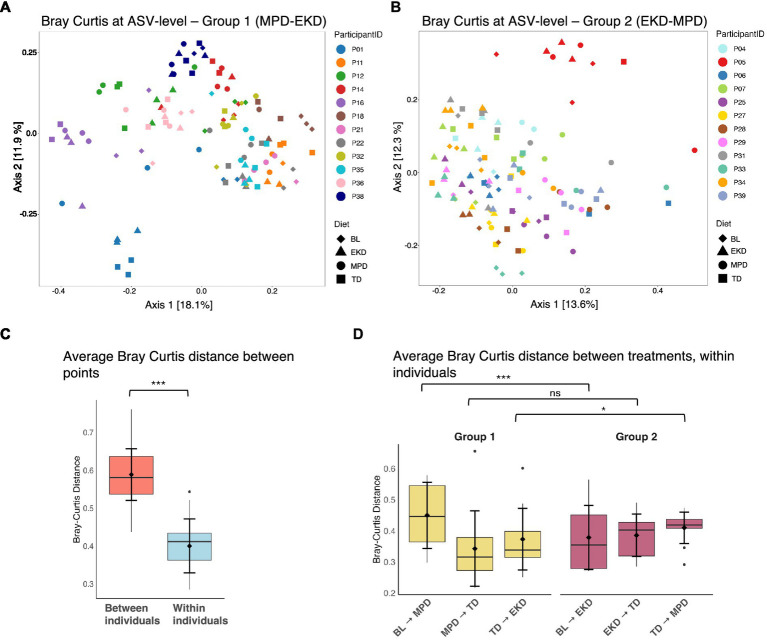
PCoA of Bray–Curtis dissimilarity at ASV level coloured by participant (sample-level, *n* = 231 samples), grouped by treatment group with **(A)** being group 1 individuals and **(B)** being group 2 individuals; **(C)** Boxplot comparing average Bray–Curtis distance between individuals versus all points within each individual, with Wilcoxon test (^***^p < 0.001); **(D)** Boxplot comparing average Bray–Curtis distance between sequential treatments within each individual, with Wilcoxon test comparing treatments by group (^*^p < 0.05, ^***^p < 0.001).

To further explore diet effects and factors associated with species turnover, we used beta diversity metrics to analyse changes in microbiome composition. Analysis of Bray–Curtis dissimilarity at the ASV level demonstrated significantly lower within-individual dissimilarity over time compared to between-individual dissimilarity (Figure 3C). This ‘individuality’ of microbiomes was more visible in PCoA plots of Group 1 than Group 2 (Figures 3A,B). The higher explained variation and clearer between-individual separation are consistent with greater initial between-individual differences in Group 1. In support of this, the average Bray–Curtis distance between points from different individuals was significantly higher in Group 1 than Group 2 (*p* < 0.001) (Supplementary Figure 7B). Such starting community effects may have been further amplified by community turnover associated with the diet transition sequence. Although alpha diversity was higher on MPD, this diet was also associated with greater within-individual community turnover. In both treatment groups, within-individual dissimilarity was highest when the dogs were transitioned to the MPD. In Group 1, the initial transition from baseline to MPD resulted in greater within-individual dissimilarity than the baseline-to-EKD transition in Group 2 (Figure 3D; *p* < 0.001). Conversely, Group 2 showed greater within-individual dissimilarity when switched to MPD (*p* < 0.05). Individual-specific abundance patterns across diets are shown in Supplementary Figure 8.

To assess whether baseline microbiomes could be grouped into community types [“enterotypes”; sensu Costea et al. (66)], we performed PAM clustering of baseline samples (24 dogs, 1–3 per dog) across multiple taxonomic levels. Two clusters were identified at both ASV- and family-levels by the Calinski–Harabasz index (Supplementary Figure 9), and dogs with multiple baseline samples were consistently assigned to the same cluster (16 in Cluster 1, 6 in Cluster 2; Supplementary Figures 10A,B). However, these community types were not stable across dietary interventions: of the six dogs predominantly in Cluster 2 at baseline, only two retained this assignment, while three dogs initially in Cluster 1 shifted to Cluster 2 (Supplementary Figures 10C,D). Indicator ASVs also differed between baseline-only and all-sample clustering, further indicating that cluster-defining features were not conserved following dietary change (Supplementary Figures 10E,F). Thus, although microbiomes showed strong individuality, baseline-defined community type assignment was not consistently maintained following dietary intervention.

### Diet selected for distinct community states across individuals over time

**Figure 4:**
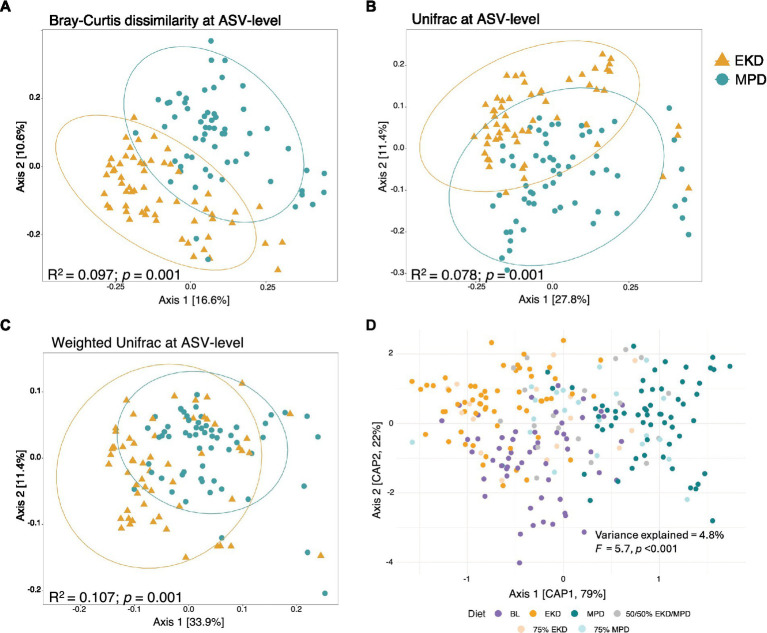
Beta diversity analysis of microbiome data at the ASV level across EKD vs. MPD diet treatments (all samples). **(A–C)** Display the Bray–Curtis dissimilarity, unweighted Unifrac, and weighted UniFrac distances, respectively, visualised using Principal Coordinate Analysis (PCoA) with samples clustered according to diet group (EKD vs. MPD, *n* = 119 samples). Orange triangles represent EKD samples and green circles represent MPD samples. Pairwise comparison tests were performed between the diet groups using Adonis. **(D)** Distance-based redundancy analysis by capscale (db-RDA) showing the relationship between community composition (Hellinger-transformed Bray–Curtis dissimilarity) and proportion of MPD vs. EKD (on a scale of 0–1) as the explanatory variable, with TD samples split according to transition sequence (*n* = 231 samples). Permutation ANOVA was used to determine the strength of the model (999 permutations).

We subsequently explored the effect of the MPD and EKD diets as drivers of microbiome composition. PCoA analyses based on Bray–Curtis dissimilarity, unweighted UniFrac, and weighted UniFrac demonstrated strong separation of samples by diet at the ASV level. Distinct diet-induced community structure was supported by pairwise comparison tests for all three plots (*p* = 0.001), with the explained variation highest for weighted Unifrac (Figures 4A–C).

To measure the contribution of diet to microbiome composition, db-RDA models based on Bray–Curtis dissimilarity were used to assess the relationship between microbiome composition and diet, with baseline designated as the reference category. Given cohort heterogeneity in age and bodyweight, these variables were included as continuous covariates to assess their contribution to explained variance. In db-RDA analyses, diet was a significant predictor of microbiome composition in all models where it was included (12 permutation tests, *p* = 0.001), explaining more constrained variance than age or bodyweight (Figure 4D; Supplementary Figure 11). In the two models including diet, EKD and MPD samples were robustly separated along the CAP1 axis. Separation along CAP1 was strongest in the diet-alone model (79% of constrained variance) than the diet + covariates model (43%), although total explained variance increased modestly with inclusion of age and bodyweight (4.8% vs. 8.9%). These patterns indicate that diet effects were strongly directional, whereas inclusion of covariates distributed variation more diffusely across multiple axes. This highlights both diets as major drivers of significant compositional disturbances beyond natural inter-individual variation.

### Differential abundance of microbial taxa & functional prediction

**Figure 5:**
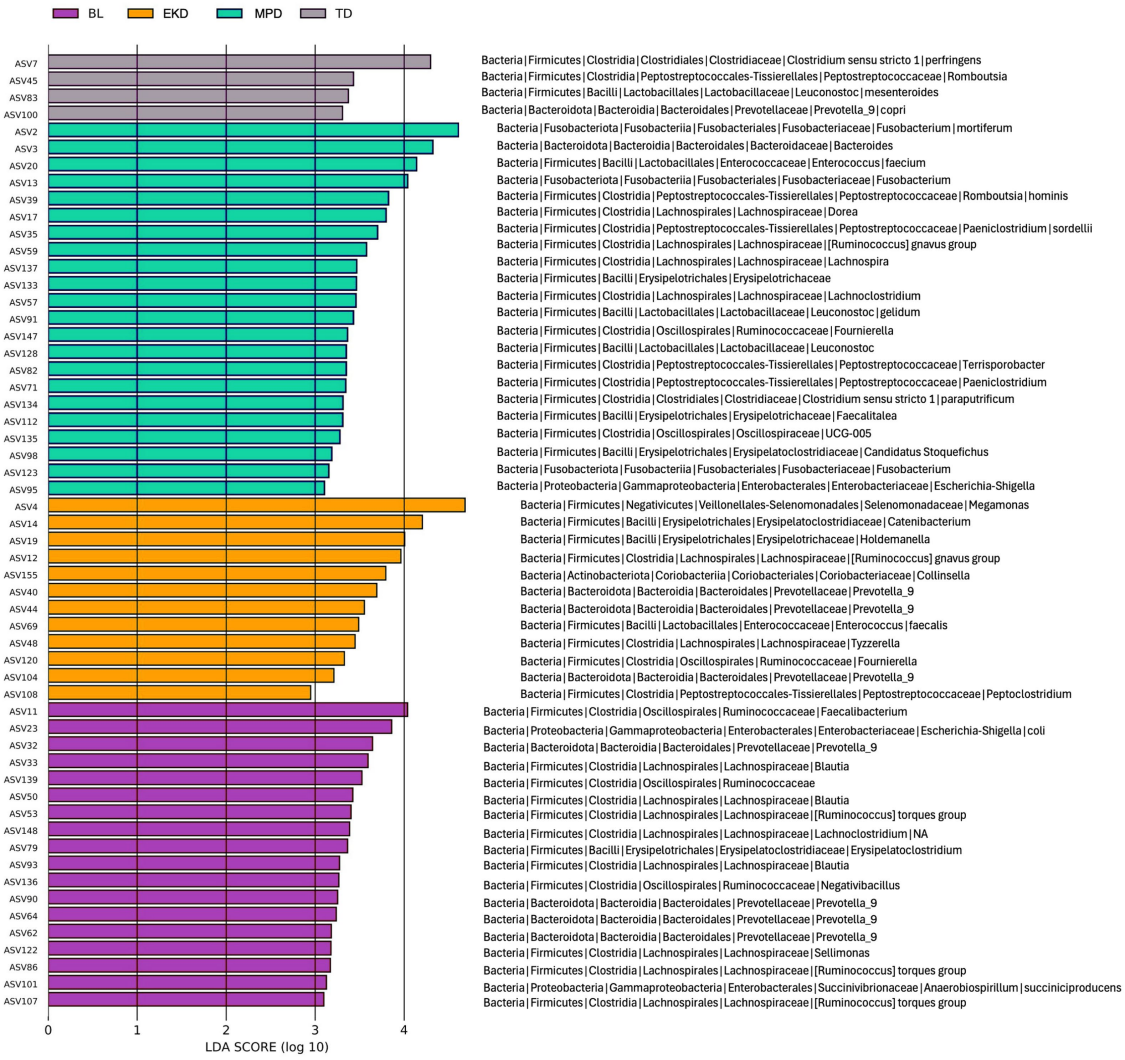
LEfSe differential abundance analysis of bacterial taxa at the ASV level across all diet conditions (BL, EKD, MPD, and TD). Analysis was performed on quality-filtered samples (*n* = 231 samples), and features with log_10_ LDA score >2 are shown.

LEfSe analysis identified microbial taxa associated with each diet treatment. A total of 47 ASVs were found to be discriminant across all diet treatments, with 20 ASVs enriched on MPD, 10 enriched on EKD, 18 enriched on BL, and 4 enriched on TD (Figure 5). Several taxonomic clades distinguished dietary groups, including Fusobacteriaceae, which were enriched when dogs were fed MPD, and Selenomonadaceae, which were enriched when dogs were fed EKD (Supplementary Figure 12A). ALDEx2 analysis comparing MPD and EKD identified eight genera significantly enriched on MPD and 10 genera significantly enriched on EKD (*p* < 0.05) (Supplementary Figure 13).

Functional potential associated with diet was assessed using PICRUSt2. Eight MetaCyc pathways differed significantly in predicted abundance between EKD and MPD (LDA > 1, *p* < 0.05) (Supplementary Figure 14A). These largely included amino acid metabolism and biosynthesis pathways. All significantly different pathways showed higher predicted abundance on EKD vs. MPD, though this pattern may partly reflect reduced diversity observed on EKD. Using the KEGG database, 96 KOs differed significantly in predicted abundance between EKD and MPD (LDA > 1, *p* < 0.05). These KOs were grouped by KEGG pathway to evaluate broader patterns (Supplementary Figure 14B). Most pathways were enriched on the EKD compared to MPD and BL, whereas seven pathways were higher in abundance on MPD. Overall, predicted functional profiles differed between diets, consistent with diet-associated taxonomic shifts inferred from ASV composition.

## Discussion

Foods are complex mixtures of nutrients that vary in structure, digestibility, and composition. The distribution of different nutrient sources within a food, their chemical modification, and their rates of absorption can all influence animal physiology. Significantly, animals can adaptively respond to changes in food sources or nutrient availability to maintain physiological homeostasis. The gut microbiome can modulate these processes through microbial metabolites and microbial-associated molecular patterns (MAMPs) that influence host regulatory pathways, potentially driving either adaptive (health) or maladaptive outcomes (dysbiosis) (10, 13, 17). Most diet–microbiome studies have focused on either compositional aspects of food such as nutrient contents, macronutrient distribution, and energy density, or variation in food intake rates and their effects on host health (67–70). While commercial extruded dog foods and human ultra-processed foods differ in ingredients and regulatory context, they share many processing features, including high-temperature extrusion, refined raw materials, and structural modification of nutrients. This motivates the need to explore the suite of biological effects that may differ between such foods.

Despite recent attention on associations between UPF intake and chronic disease in humans, understanding of the impact of food processing methods *per se* on diet–microbiome interactions remains limited. Available data indicate that health risk from UPFs is primarily associated with nutrient-imbalanced discretionary foods. However, currently, little is known about how the consumption of highly processed, yet nutritionally complete, foods affects host outcomes. This question is particularly important in pet dogs who are frequently fed highly processed foods as their primary diet (e.g., ‘wet’ canned or ‘dry’ extruded kibbles). Such foods are convenient and are formulated to be complete and balanced with respect to nutritional requirements. However, production processes involve high-temperature, high-pressure treatments that alter food chemistry, structure, and nutrient availability. Such changes have the potential to differentially impact digestibility and thereby microbial influence on (mal)adaptive host responses to diet.

Our study tested two nutritionally complete foods as staple diets for overt differences in microbial or physiological responses with the potential to drive distinct outcomes. We used commercially available products matched for their capacity to meet maintenance energy and essential nutrient requirements, but differing by production methods. The minimally processed test food differs from the broadly typical macronutrient distribution, fibre content, and moisture content of dry pet dog foods. Pet food labelling regulations require reporting of crude fibre (CF) rather than total dietary fibre (TDF), and batch-matched proximate analysis of TDF was only available for the minimally processed diet. The manufacturer-declared CF values for each diet indicate higher CF in the EKD. Extruded kibble diets require sufficient starch content to enable expansion and structural stability during processing, which limits the extent to which protein proportions can be increased in a commercially viable dry format. A lower protein and higher carbohydrate content is broadly typical for dry versus wet dog foods. Our MPD test food lies significantly outside the range of ratios for protein:carbohydrate (as sources of metabolisable energy) reported from dry kibbles (Supplementary Figure 2A) and is at the limits of the reported range for macronutrient distributions in wet foods (Supplementary Figure 2B). Our test diets thus differ in multiple dimensions but are broadly representative of two types of foods commonly fed to pet dogs. As a prelude to the assessment of outcomes of long-term feeding, we compared physiological and microbiome responses under realistic pet dog conditions.

Immuno-metabolic outcomes in canine dietary studies are commonly assessed by measurement of various cytokines and pancreatic hormones. Appetite- and gut-secreted hormones are less frequently reported and, when assessed, these hormones typically show minimal dietary effects (71). Much of the existing work has therefore focused on markers interpreted primarily as indicators of metabolic dysfunction (e.g., insulin), rather than hormones involved in appetite regulation. However, changes in feeding behaviour are now recognised as adaptive responses to altered nutrient availability and potentiators of physiological change. We found statistically significant differences between MPD- vs. EKD-fed dogs for mean fasting and post-prandial concentrations of GIP and PYY, and in average post-prandial blood glucose concentrations. Furthermore, GLP-1, insulin, amylin, and active ghrelin also showed substantially lower mean values in MPD-fed dogs, albeit not significant at *p* < 0.05. Because many of these hormones originate from enteroendocrine cells distributed along the intestinal epithelium, they represent physiological pathways through which diet composition and gut microbial activity can influence host metabolic regulation.

The post-prandial measurements primarily reflect acute responses to meal composition and digestion. Ethical constraints associated with a minimally invasive design meant that blood samples of sufficient volume for hormone analysis could only be taken at two time points. This limits the ability to detect the peak or nadir of certain hormones, which can vary by individual and hormone type. Further caveats for interpretation are that dogs were fed their standard meal portion of the test diet, and some individuals did not complete the meal; compositional and intake differences of the diets will contribute to post-prandial differences.

Fasting measurements (*T* = 0 h) are less subject to these factors, and we interpret these as putative indicators of early physiological differences in adaptive responses to each diet. Although circulating hormone concentrations are influenced by multiple interacting factors and should be interpreted cautiously, a significant factor is the state of the secreting cell populations. We note that hormones from the same cell types had consistent outcomes. Of the hormones where we observed differences, active ghrelin is secreted by X/A-like cells in the stomach; insulin and amylin are secreted from beta cells in the pancreas, whereas GIP is primarily secreted by K cells and PYY and GLP-1 by L cells in the small intestine in dogs. The fasting concentrations of these hormones were reduced in MPD-fed dogs. We postulate that the differences are feasibly the result of short-term physiological responses to the diets, since both diet composition and microbial activity are known to influence differentiation and function of enteroendocrine cells (EECs) (72). Dietary substrates reaching the intestine and microbially derived metabolites can alter EEC abundance and signalling capacity, while microbial-associated molecular patterns (MAMPs) provide additional regulatory inputs affecting hormone secretion (10, 13, 72). These differences in fasting concentrations plausibly result from early adjustment of these diet–microbiome–host signalling pathways and may indicate the potential for longer-term changes. Confirmation of this hypothesis requires direct evidence, but the observations align with prior evidence that dogs are physiologically sensitive to nutrient composition.

Across the study, significant differences in immuno-metabolic health outcomes attributable to the diets were not observed. We detected a few changes in immune function between the diets compared to other studies (73), which may reflect assay sensitivity or simply that changes did not occur within our study timeframe. Mean bodyweight did not differ significantly between baseline and the final timepoint. However, in both treatment groups, the introduction of MPD was associated with a small decrease in average bodyweight, whereas the introduction of EKD was associated with a small gain in weight. Because of this directional pattern, the sequence in which dogs transitioned from MPD to EKD (Group 1) resulted in a statistically detectable increase in mean bodyweight across that phase of the study. A significant difference in average blood glucose response between the two diets was also observed within treatment groups, although this response varied substantially between individuals and may partly reflect meal differences. Other studies have also noted variability in canine metabolic and hormone responses to different diets (74, 75). Differences between individuals in meal completion at clinic, breed (76), age (77), body condition (78–80), and, potentially, the dogs’ prior dietary histories may all affect post-prandial blood glucose responses. Our findings thus demonstrate that both diets supported maintenance of health during short-term feeding, while differences in fasted-state endocrine outcomes highlight the potential for longer-term feeding to drive different physiological outcomes.

The gut microbiome is known to respond rapidly to dietary change and can influence enteroendocrine signalling through microbial metabolites and associated molecular signals (72). Prior studies in dogs (81–88) report microbiome shifts within short periods; therefore, we expected to see microbiome change here. The longitudinal sample design with repeat samples in four experimental windows (baseline, diet 1, transition, and diet 2) facilitated resolution of treatment-induced shifts in alpha diversity metrics. We found the mean ASV richness of individuals when fed EKD was significantly lower than baseline, MPD, and during diet transition, but note the effect was greater when dogs were fed EKD after MPD (with a smaller effect when EKD was fed first). This may reflect that the baseline diet of most dogs was dominated by a kibble food, such that introduction of EKD represented a modest dietary change, relative to the MPD.

Exploration of between-individual microbiomes revealed that the two test diets had a determinative effect on community structure, with significant differences between microbiomes of dogs fed the two diets (three independent samples per dog for each diet). However, the major driver of variance across the complete microbiome dataset was the individual dog. The average Bray–Curtis distances for the 12 samples from the same dog were significantly lower than those between dogs. Such individuality is commonly seen in mammalian microbiome studies, and the potential of historical conditions (hysteresis) to influence microbiome response to change is widely postulated to contribute to variable outcomes to dietary interventions (89). This observation motivated our subsequent explorations of whether specific taxa consistently responded to the different diets across individuals or if baseline communities could be grouped into broader community states that might influence the magnitude and direction of microbiome shifts following dietary change (89–91).

Differential abundance analysis revealed distinct microbial taxa associated with each diet. On MPD, LEfSe analysis identified enrichment of *Fusobacterium* ASVs (ASV2 and ASV13) and *Enterococcus faecium* (ASV20), which was corroborated at the genus level by ALDEx2. Enrichment of *Fusobacterium* on MPD aligns with its known preference for amino acids derived from dietary proteins (92) and with reports of increased abundance in dogs fed foods that are more gently cooked and higher in protein (88, 93, 94). Similar associations have been reported for *E. faecium* (95). In contrast, EKD was characterised by enrichment of ASV4 (*Megamonas*), ASV14 (*Catenibacterium*), ASV19 (*Holdemanella*), and multiple ASVs assigned to the unclassified genus *Prevotella_9*—all of which were also supported at the genus level. These taxa are commonly associated with carbohydrate-rich and extruded kibble diets in canine dietary studies (95, 96). Consistent directional shifts in the abundance of these ASVs across dogs further support the robustness of these diet-associated patterns. Predicted genetic traits associated with these taxa (PICRUSt2 output) were consistent with diet driving increased propanoate on EKD (linked to higher carbohydrate availability) and increased pyruvate on MPD (linked to higher protein availability).

Microbiome composition is particularly shaped by total dietary fibre content as well as fibre source, physicochemical properties, and fermentability (97–99). Here, both diets provided mixed soluble and insoluble fibre sources, but MPD is expected to have lower TDF (although TDF data were not available for the EKD diet formulation used). The differential taxa did not include species known to be fibre-specialists, suggesting that fibre quantity or quality was unlikely to be the primary driver of the observed microbiome differences. This supports the interpretation that macronutrient distribution is the key determinant of diet-associated microbial responses in this study.

We applied *de novo* PAM clustering to baseline community types (66). Such community typing does give recurrently observed patterns in humans (enterotypes) based on counter-variation in *Prevotella* and *Bacteroides,* but both the number of definable enterotypes and how to define them are debated. Here, the community type assignations of individuals from baseline samples did not remain consistent after dietary interventions. While baseline variation (and individual history) may still contribute to individual trajectories, our results suggest that response is primarily driven by dietary treatment. We could not observe any predictive association with community types within our small cohort; however, it is possible that larger canine datasets may reveal baseline “enterotypes” with greater predictive value, response patterns, or associated health outcomes.

Our study contributes to the growing body of research into dog food alternatives to extruded kibble diets (100, 101) and complements increasing interest (from both dog owners and researchers) in nutrition optimisation for pet dogs (1, 27, 102). We assessed the response to two staple foods in typical owner-maintained pet dogs. A secondary aim was to identify microbiome factors that may influence or predict the outcomes from long-term feeding. Such data are valuable to inform the design of clinical trials assessing the effectiveness of diets for supporting healthspan in pet dogs. We observed significant differences in average microbiome and physiological responses elicited by MPD versus EKD in a short-term intervention. EKD represents commonly fed kibbles and is not nutrient-matched to MPD, because differences in macronutrient architecture systematically co-occur with processing in real-world formulations. As shown in the right-angled mixture triangle analysis in Supplementary Figure 2, MPD occupies a higher protein, lower carbohydrate region relative to a subset of commercial kibbles. Expressing these data as macronutrient ratios (P:C and P:F) emphasises that the displacement of MPD reflects a shift in macronutrient balance rather than a single nutrient.

Our study demonstrates the feasibility of assessing microbiome and physiological responses to diet in a well-characterised canine cohort and highlights the feasibility of in-community studies for interrogating diet–microbiome–host interactions. Such studies may therefore contribute to improved understanding of nutrition-related disease risk and prevention in pet dogs, while also offering insights into mechanisms of diet and microbiome interaction for animal health. Functional predictions based on 16S rRNA assignment to taxa with complete genomes permit useful hypothesis development, but integration with metagenomic or metabolomic approaches would allow direct assessment of microbial metabolic and diet interactions. Because pet dog cohorts are less tightly experimentally controlled and are genetically and physiologically diverse, causal mechanisms cannot be definitively attributed to dietary nutrient composition or processing level. There is scope for future studies to improve on this by incorporating experimental diets with controlled macronutrient profiles and fibre composition, longer feeding periods, and more homogeneous cohorts. Such studies will be important for assessing the relative contributions of different diet dimensions to pet dog healthspan.

## Data Availability

The datasets presented in this study can be found in online repositories. The names of the repository/repositories and accession number(s) can be found at: https://www.ebi.ac.uk/ena, PRJEB96190 https://zenodo.org/, 10.5281/zenodo.15844682.
