# Supplementary material for: Diet-induced metabolic and faecal microbiome responses in pet dogs fed a minimally processed versus extruded kibble diet
Source: Front Vet Sci. 2026 Apr 10;13:1734572. doi: 10.3389/fvets.2026.1734572 (PMC13107655; doi:10.3389/fvets.2026.1734572)
Supplement: Supplementary file 1 [file Supplementary_file_1.zip › Supplementary Material/Supplementary file 1.docx]

# Supplementary Materials


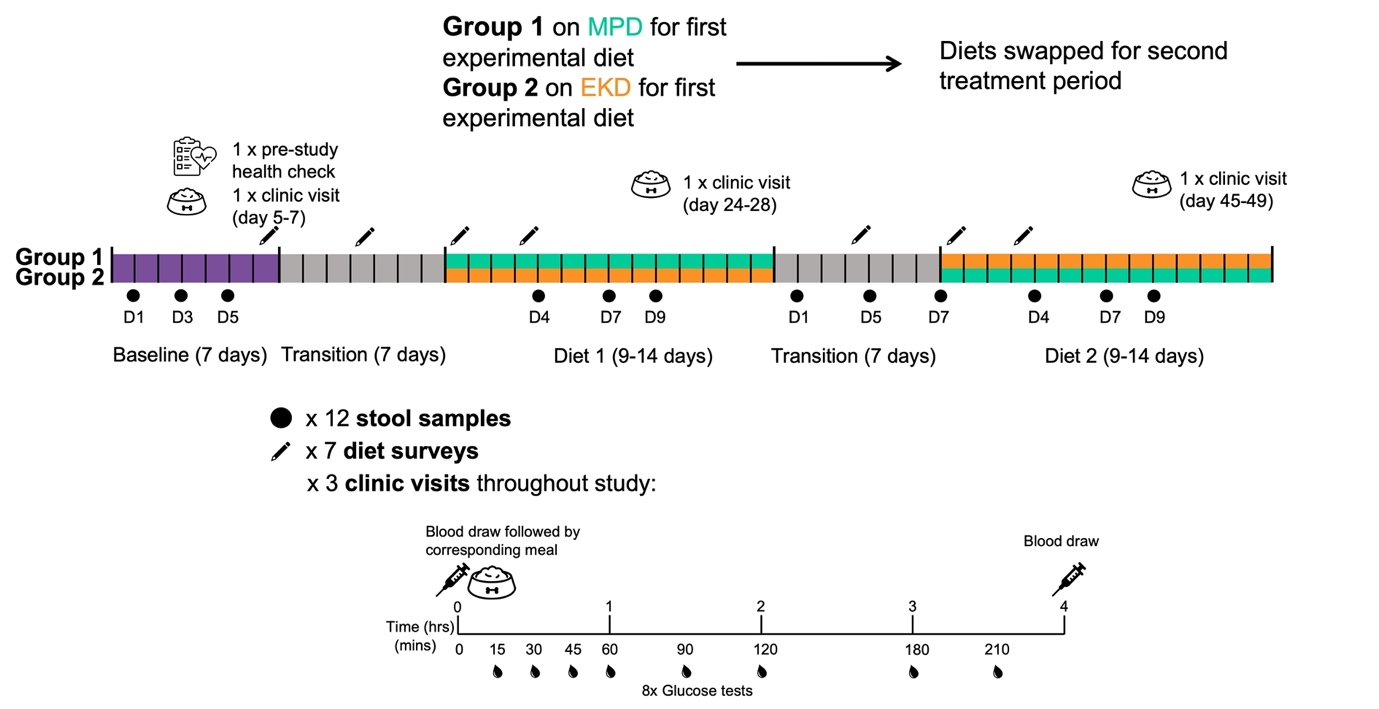


#### Supplementary figure 1: study treatment sequences and sampling timepoints, including 12 stool sample timepoints (and corresponding faecal consistency scores), 7 diet surveys (which consisted of 24-hr recall of food consumed by the dog), in-clinic blood and additional blood glucose sampling (fasting and post-prandial). Transition periods involved a gradual shift in the proportion of the previous and following meals, 3 days of 75:25; then 2 days of 50:50, then 2 days of 25:75. Clinic visits were scheduled as close as possible to the last day of the test diet period and always after a minimum of 9 days on the test diet. Visits were conducted in the morning to ensure dogs had been fasting for a minimum of 12 hours. Owners received reminders to collect stool samples and complete surveys on specified days, but adherence to the timeline varied.


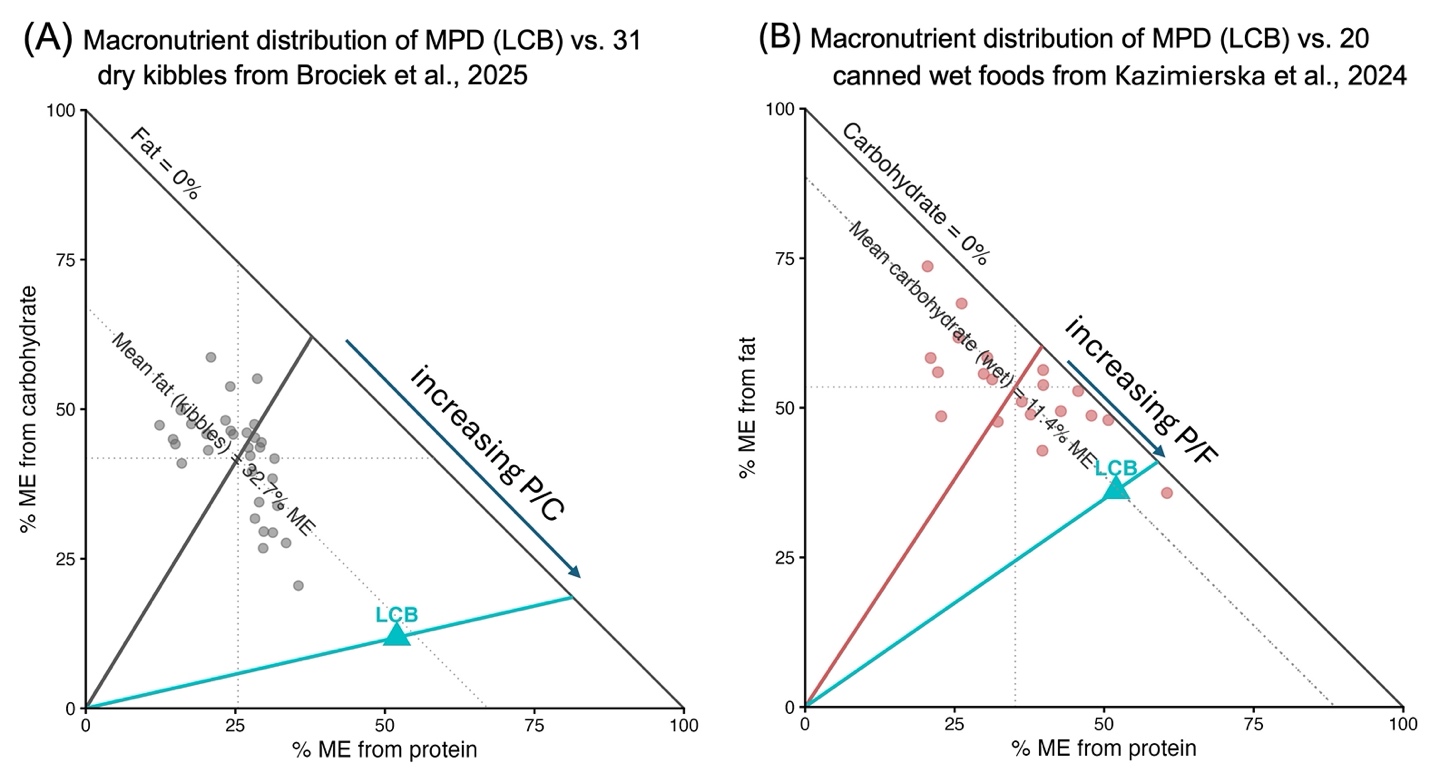


Supplementary figure 2: Macronutrient energy composition of minimally processed diet (MPD; Lyka Chicken Bowl, LCB) relative to published commercial pet foods *Percent metabolizable energy (%ME) from protein and either carbohydrate (A) or fat (B) are shown on orthogonal axes. The third macronutrient is represented along the negative diagonal isoclines, where values increase from 0% at the boundary (protein + carbohydrate/fat = 100%) toward 100% at the origin*. (A) Distribution of 31 extruded dry kibbles (Brociek et al., 2025) plotted on protein (%ME) versus carbohydrate (%ME) axes. (B) Distribution of 20 commercially available wet canned dog foods (Kazimierska et al., 2024) plotted on protein (%ME) versus fat (%ME) axes. Grey (A) and red (B) points represent individual products within each published dataset. The teal triangle indicates Lyka Chicken Bowl. Thin internal lines denote the mean macronutrient energy proportions of each reference set. The solid diagonal in each panel indicates the boundary where the third macronutrient equals zero. Directional arrows illustrate increasing protein-to-carbohydrate (A) and protein-to-fat (B) ratios. These reference distributions are drawn from published datasets and are intended to provide contextual bounds for comparison rather than represent market-wide prevalence.

#### Supplementary table 1A. Nutritional description of Hill’s Science Diet Healthy Mobility as provided by the manufacturer. Direct comparison of nutritional content of two diets difficult to ascertain due to differences in manufacturer reporting.

| **Nutrient** | **Dry Matter (%)** |
| --- | --- |
| Energy | 3617 Kcal/kg |
| Protein | 22.4 |
| Fat | 14.7 |
| Carbohydrate / NFE | 54.2 |
| Crude Fiber | 2.4 |
| Calcium | 0.99 |
| Phosphorus | 0.69 |
| Potassium | 0.91 |
| Sodium | 0.3 |
| Magnesium | 0.128 |
| Vitamin C | 202 ppm |
| Vitamin E | 708 IU/kg |
| Total Omega-3 FA | 1.29 % |
| Total Omega-6 FA | 3.77 % |
| Glucosamine | 644 ppm |

#### Supplementary table 1B. Nutritional description of Lyka Chicken Bowl as provided by the manufacturer. Direct comparison of nutritional content of two diets difficult to ascertain due to differences in manufacturer reporting.

| **Nutrient** | **Dry Matter (%)** |
| --- | --- |
| Energy | 1182 Kcal/kg |
| Protein | 65 |
| Fat | 12 |
| Carbohydrate / NFE | 11 |
| Crude Fiber | 4 |
| Calcium | 1.4 |
| Phosphorus | 0.7 |
| Potassium | 0.7 |
| Magnesium | 1 g/kg |
| Linoleic Acid | 2 |
| Choline | 1618 mg/kg |
| Folate, DFE | 1817 mcg/kg |
| Niacin | 242 mg/kg |
| Pantothenic Acid | 40 mg/kg |
| Riboflavin | 9 mg/kg |
| Thiamin | 9 mg/kg |
| Vitamin A, RAE | 10508 mcg/kg |
| Cobalamin | 0 mg/kg |
| Pyridoxine | 13 mg/kg |
| Alpha Tocopherol (Vitamin E) | 192 IU/kg |
| CA:P Ratio | 1.2 |
| Chloride | 67 mg/kg |
| Copper | 21 mg/kg |
| Iodine | 2 mg/kg |
| Iron | 154 mg/kg |
| Manganese | 12 mg/kg |
| Selenium | 0.4 mg/kg |
| Zinc | 151 mg/kg |
| Vitamin D2 + D3 | 1939 IU/kg |
| EPA + DHA | 0.40 |

#### Supplementary table 1C. Ingredients as provided by the manufacturers for Hill’s Science Diet Healthy Mobility and Lyka Chicken Bowl.

|  | **Ingredient list** |
| --- | --- |
| Hill’s Science Healthy Mobility | Brewers Rice, Chicken Meal, Whole Grain Sorghum, Brown Rice, Whole Grain Wheat, Chicken Fat, Cracked Pearled Barley, Soybean Meal, Chicken Liver Flavor, Dried Beet Pulp, Oat Fiber, Pork Liver Flavor, Lactic Acid, Potassium Chloride, Iodized Salt, DL-Methionine, Choline Chloride, L-Lysine, Calcium Carbonate, vitamins (Vitamin E Supplement, Niacin Supplement, Thiamine Mononitrate, Calcium Pantothenate, Vitamin A Supplement, Riboflavin Supplement, Biotin, Vitamin B12 Supplement, Pyridoxine Hydrochloride, Folic Acid, Vitamin D3 Supplement), minerals (Ferrous Sulfate, Zinc Oxide, Copper Sulfate, Manganese Sulfate, Calcium Iodate, Sodium Selenite), Taurine, Mixed Tocopherols for freshness, Natural Flavors, Beta-Carotene. |
| Lyka Chicken Bowl | *Chicken Breast, Butternut Squash, Chicken Heart, Carrot, Chicken Liver, Cauliflower, Spinach, Egg, Psyllium Seed Husk, Fish Oil, Shiitake Mushroom Powder, Kelp, Celtic Sea Salt, Flaxseed Oil, Basil, TriCalcium Phosphate, Vitamin E Oil, Iron Chelate, Zinc Chelate, Copper Chelate, Manganese Chelate, Vitamin D3, Vitamin B1.* |


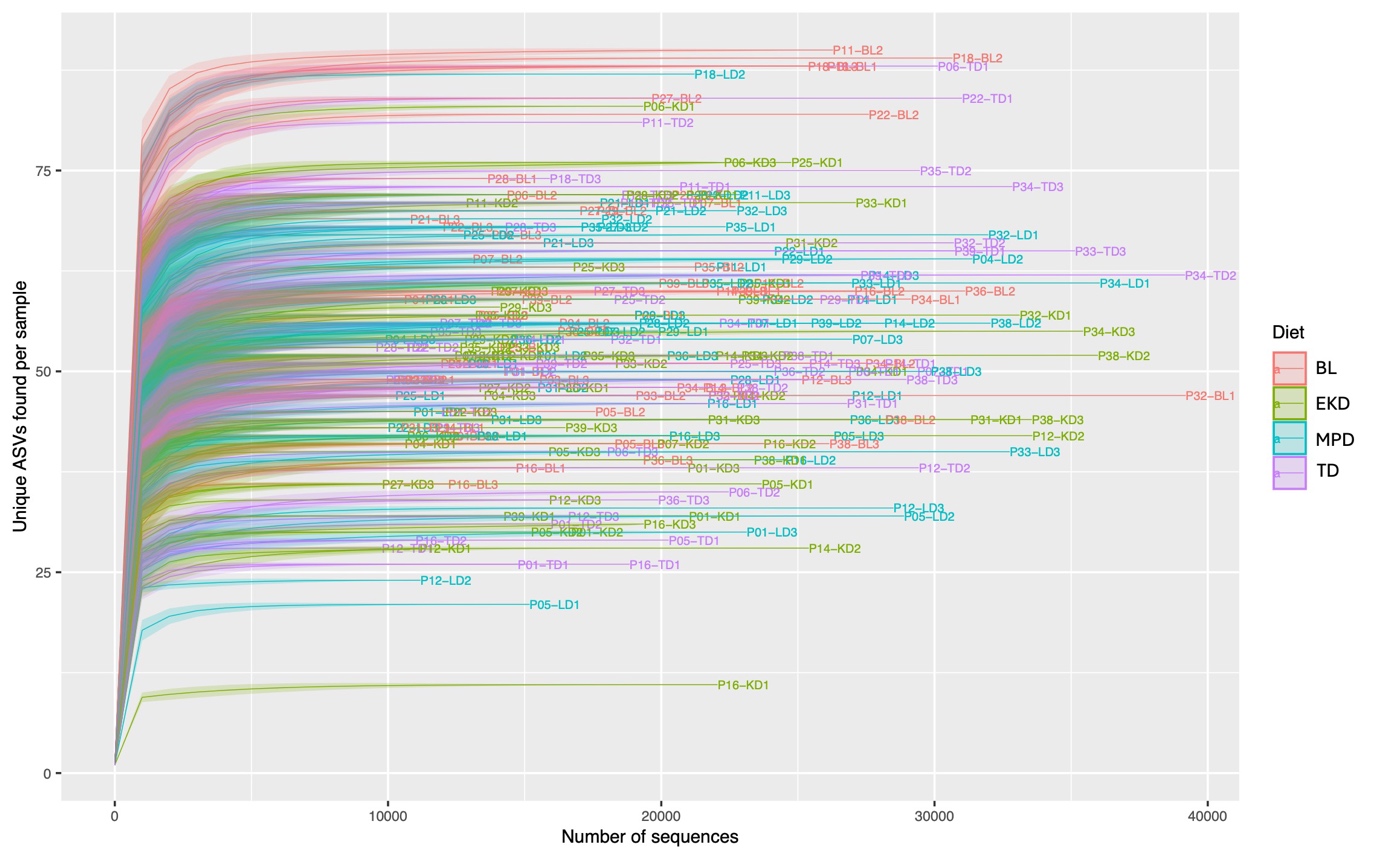


#### Supplementary figure 3: Rarefaction curves for all samples following 16S ASV sample filtration using DADA2 pipeline (n = 231 samples).

#### Supplementary table 2A. Metadata and biometrics for all participants in the study. BCS = body condition score, BWT = bodyweight. Participants P04 & P05, P27 & P28 and P35 & P36 were cohabitants. *Asterisk denotes measurements not taken (reasons described in Methods).

| *Participant ID* | *Group* | *Age* | *Sex* | *Breed* | *BCS (at Baseline)* | *Main Diet at Baseline* | *Study completion* | *BWT at Baseline (kg)* | *BWT after MPD*  *(kg)* | *BWT after EKD*  *(kg)* |
| --- | --- | --- | --- | --- | --- | --- | --- | --- | --- | --- |
| P01 | 1 | 2 | F | Golden Retriever | 5 | Kibble | Yes | 27 | 26.7 | 27.1 |
| P11 | 1 | 4 | F | Greyhound | 5 | Kibble and carrot/other treats | Yes | 30.4 | 29.4 | 31.3 |
| P12 | 1 | 6 | M | Greyhound | 6 | Beef mince, chicken breast, kibble | Yes | 31 | 29.8 | 30 |
| P14 | 1 | 2 | M | Mini American Shepherd | * | 50% kibble, 25% air-dried raw, 25% cooked dog food roll or homecooked fresh diet | Yes | 15.2 | * | * |
| P16 | 1 | 9 | M | American Bulldog mix | 5 | 2/3 Kibble/ 1/3 Raw | Yes | 43.1 | 43.1 | 43.8 |
| P18 | 1 | 9 | F | English Staffordshire Terrier | 5 | Kibble and occasional carrot/chewstick | Yes | 20 | 20.2 | 20.4 |
| P21 | 1 | 2 | M | Labrador | 4 | Kibble | Yes | 22.5 | 21.5 | 23 |
| P22 | 1 | 2 | F | Labrador | 4 | Kibble | Yes | 17.8 | 17.8 | 19.2 |
| P32 | 1 | 5 | F | Staffordshire Terrier x Jack Russell | 5 | Kibble and canned dog food; occasional raw chicken drumsticks/other treats | Yes | 21.4 | 20.3 | 20.1 |
| P35 | 1 | 4 | F | Labradoodle | 7 | Kibble | Yes | 18.1 | 18.7 | 20.1 |
| P36 | 1 | 4 | M | Cavoodle | 5 | Kibble | Yes | 13 | 13.1 | 13.6 |
| P38 | 1 | 2 | M | Jack Russell x Australian Terrier | 6 | Wet food & dry food | Yes | 11 | 11.3 | 11 |
| P04 | 2 | 4 | F | Samoyed | 6 | Kibble | Yes | 27 | 27.4 | 27.6 |
| P05 | 2 | 6 | M | Japanese Spitz | 5 | Kibble | Yes | 12 | 11.6 | 12.1 |
| P06 | 2 | 4.5 | M | Wolfhound x Kelpie | 5 | Kibble | No | 30.4 | * | 31.8 |
| P07 | 2 | 2.5 | M | Nova Scotia Duck Tolling Retriever | 7 | Kibble | Yes | 26.4 | 26.5 | 27.4 |
| P25 | 2 | 5 | F | Border Collie | 4 | Mixture of commercial minimally-processed dog foods and home-cooked meals | Yes | 15.5 | 15.3 | 15 |
| P27 | 2 | 3 | M | Sheepadoodle | 5 | Kibble and some food scraps | Yes | 20.9 | 19.8 | 20 |
| P28 | 2 | 3 | F | Sheepadoodle | 5 | Kibble and some food scraps | Yes | 29.1 | 27.3 | 28.5 |
| P29 | 2 | 2 | F | Border Collie | * | Kibble | Yes | 23.6 | 24.8 | 23.8 |
| P31 | 2 | 8 | M | Cavoodle | 6 | Kibble | Yes | 10.9 | 11.4 | 11.3 |
| P33 | 2 | 3 | F | Labrador | 6 | Kibble, carrot treat, peanut butter, table scraps | Yes | 35.1 | 35.2 | 35.4 |
| P34 |  | 6 | F | Bull Arab | 6 | Kibble, some berries, sardines, bread | Yes | 40.1 | 41.5 | 39.8 |
| P39 | 2 | 5 | F | Staffordshire Terrier mix | * | Kibble | Yes | 26.5 | 24.5 | 26.2 |

#### Supplementary table 2B. Summary of study cohort metadata, including Body Condition Score (BCS), Bodyweight and Age mean ± SD. All baseline (BL) metadata were available for all recruited dogs.

|  | *N total* | *N females* | *N males* | *BCS (mean, at BL)* | *BCS range* | *Bodyweight (mean kg, at BL)* | *Bodyweight range (kg)* | *Age (mean)* | *Age range* |
| --- | --- | --- | --- | --- | --- | --- | --- | --- | --- |
| ***Group 1*** (MPD-EKD) | 12 | 6 | 6 | 5.18 ± 0.87 | 4-7 | 22.54 ± 9.07 | 11-43 | 4.25 ± 2.60 | 2-9 |
| ***Group 2***  (EKD-MPD) | 12 | 7 | 5 | 5.5 ± 0.85 | 4-7 | 24.91 ± 8.94 | 11-40 | 4.33 ± 1.76 | 2-8 |
| ***All*** | 24 | 13 | 11 | 5.33 ± 0.86 | 4-7 | 23.54 ± 8.83 | 11-43 | 4.29  ± 2.17 | 2-9 |

#### Supplementary table 3. Paired-sample *t*-test results comparing bodyweight between baseline and treatment 1, treatment 1 and treatment 2 (end) by treatment sequence, and baseline to treatment 2 (end) across the full cohort. Only dogs with measurements at both timepoints were included in each paired comparison; one dog withdrew midway through the study and was excluded from analyses.

|  | *N* | *Mean (before)* | *Mean (after)* | *SD (before)* | *SD (after)* | *t* | *p-value* |
| --- | --- | --- | --- | --- | --- | --- | --- |
| Group 1: baseline – treatment 1 (MPD) | 11 | 23.2 | 22.9 | 9.2 | 8.9 | 3.1 | 0.1443 |
| Group 2: baseline – treatment 1 (EKD) | 12 | 24.8 | 24.9 | 8.8 | 8.9 | 0.6 | 0.563 |
| Group 1: treatment 1 (MPD) – treatment 2 (EKD) | 11 | 22.9 | 23.6 | 9.0 | 9.1 | 3.1 | 0.0107 |
| Group 2: treatment 1 (EKD) – treatment 2 (MPD) | 11 | 24.3 | 24.1 | 9.1 | 9.3 | 0.6 | 0.5841 |
| Baseline – End | 23 | 24.0 | 24.3 | 8.8 | 8.9 | 1.5 | 0.158 |

#### Supplementary table 4. Faecal consistency scores as provided by the owner per sample, with mixed effects analysis and Tukey’s multiple comparisons test. FCS values expressed as mean ± SD.

|  | *Baseline* | *EKD* | *MPD* | *Fixed effect (Diet) p-value* | *Pairwise comparisons* |
| --- | --- | --- | --- | --- | --- |
| FCS (n) | 64 | 62 | 55 |  |  |
| FCS | 2.69 ± 0.96 | 2.87 ± 1.29 | 2.24 ± 0.67 | 0.0021 | BL > MPD (*p* = 0.0047)  EKD > MPD (*p =* 0.0048) |


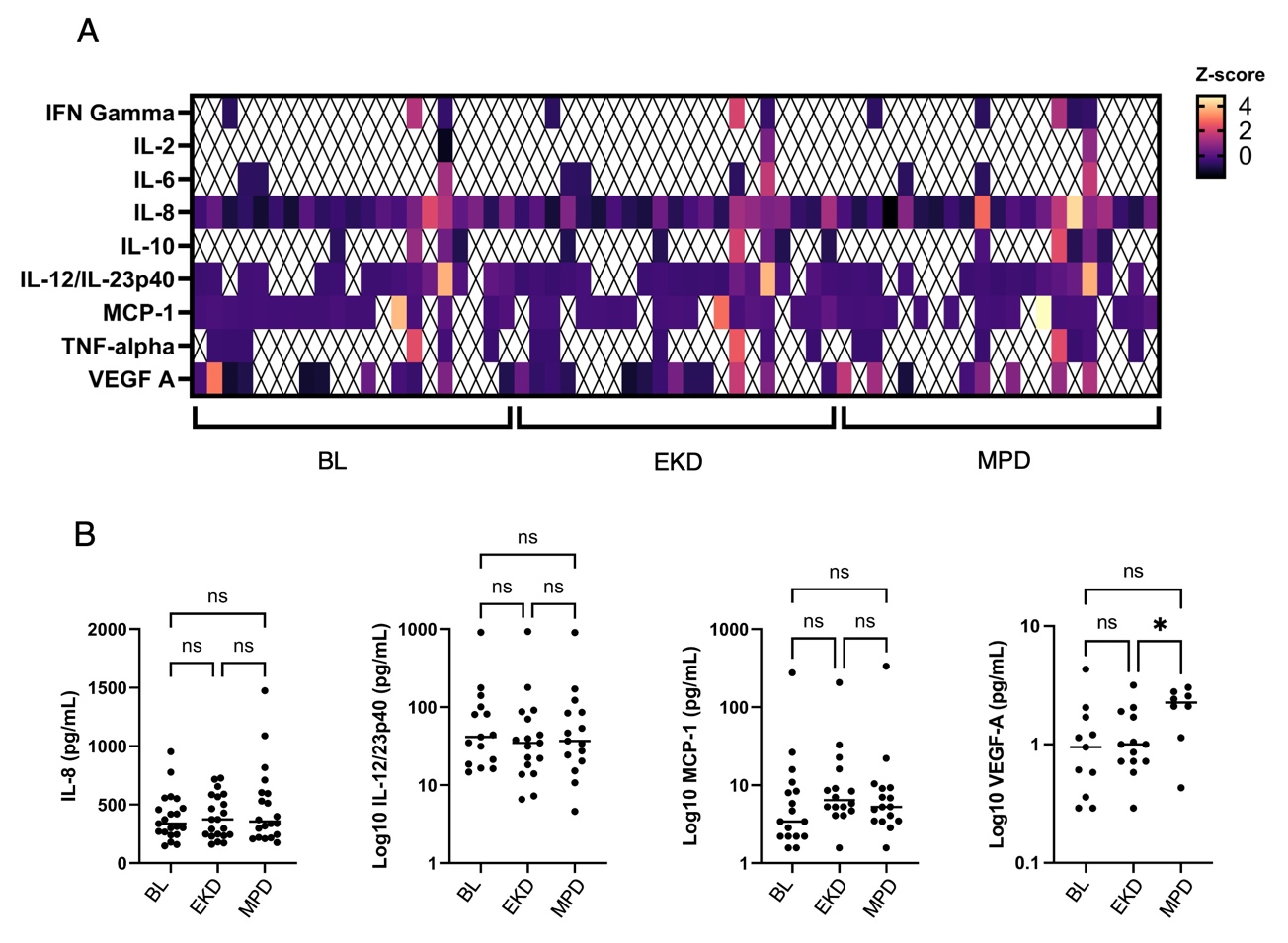


#### Supplementary figure 4: (A) Z-scored concentrations of cytokines, chemokines, and growth factors, grouped by diet (BL, EKD, MPD). Values were standardised per analyte to visualise relative differences across diets. (B) Mixed-effects models were used to test for differences in IL-8, IL-12/23p40, MCP-1, and VEGF-A concentrations between diets. Only analytes detectable in >50% of samples were included. Analyses included all dogs with available blood samples (n = 21).


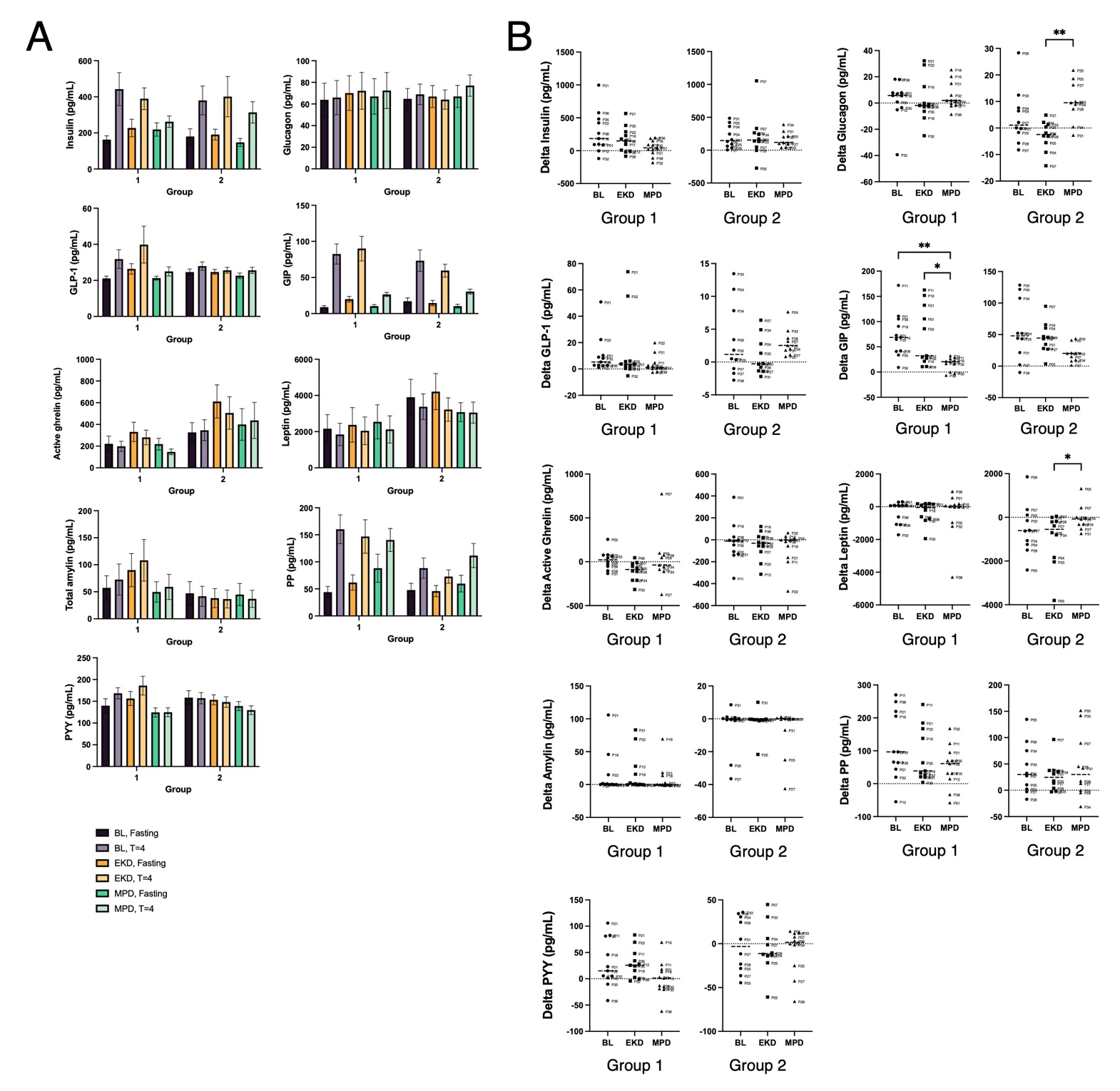


#### Supplementary figure 5: (A) Gut and appetite-related hormone concentrations according to timepoint and diet, stratified by treatment sequence (Group 1 and Group 2). (B) Within-diet changes in gut and appetite-related hormone concentrations between timepoints, plotted by treatment sequence. Data were analysed using mixed-effects models with post hoc multiple-comparisons testing; values are shown as mean ± SEM (p < 0.05; *p < 0.01; **p < 0.001). Group sizes were n = 12 for Group 1 and n = 11–12 for Group 2, reflecting one dropout during the MPD phase.

#### Supplementary table 5: Summary of circulating gut hormone concentrations measured at baseline (T0) and 4 h post-prandial (T4) during feeding of extruded kibble diet (EKD) and minimally processed diet (MPD). Values are shown as mean ± standard deviation across paired dogs (n=20 dogs, which does not include the one drop-out). Percent differences represent the within-dog relative difference between diets, calculated as EKD relative to MPD. The number of dogs showing higher concentrations on each diet is reported to illustrate inter-individual response patterns. Bolded hormones were significantly different (p < 0.05) between diets according to Figure 1 analyses.

| *Hormone* | *T=* | *Mean pg/mL on EKD (± SD)* | *Mean pg/mL on MPD (± SD)* | *% difference, EKD vs MPD* | *No. dogs higher /20* |
| --- | --- | --- | --- | --- | --- |
| Active ghrelin | T0 | 464.8 ± 410.7 | 299.8 ± 326.2 | Mean 271.7% (median 59.7%) | 13 higher on EKD |
| Active ghrelin | T4 | 387.45 ± 372.4 | 277.7 ± 361.4 | Mean 181.0% (median 22.1%) | 13 higher on EKD |
| **GIP** | T0 | 17.4± 12.4 | 10.3 ± 7.3 | Mean 197.9% (median 31.0%) | 13 higher on EKD |
| **GIP** | T4 | 75.5 ± 46.9 | 28.3 ± 9.8 | Mean 181.9% (median 138.0%) | 18 higher on EKD |
| GLP-1 | T0 | 25.6 ± 7.4 | 21.8 ± 4.0 | Mean 17.6% (median 5.3%) | 14 higher on EKD |
| GLP-1 | T4 | 33.1 ± 25.3 | 25.2 ± 6.9 | Mean 25.1% (median 3.4%) | 14 higher on EKD |
| Glucagon | T0 | 68.6± 43.4 | 66.98 ± 44.3 | Mean 2.6% (median 1.4%) | 12 higher on EKD |
| Glucagon | T4 | 68.3 ± 44.4 | 74.50 ± 44.6 | Mean -8.9% (median -13.6%) | 16 higher on MPD |
| Insulin | T0 | 209.8 ± 131.8 | 186.9 ± 105.1 | Mean 29.7% (median 7.7%) | 12 higher on EKD |
| Insulin | T4 | 395.1 ± 276.2 | 285.4 ± 140.6 | Mean 48.0% (median 30.3%) | 16 higher on EKD |
| Leptin | T0 | 3248.1 ± 3226.4 | 2782.3 ± 2499.57 | Mean 27.1% (median 29.6%) | 15 higher on EKD |
| Leptin | T4 | 2604.8 ± 2334.1 | 2541.4 ± 2180.9 | Mean 19.7% (median 22.0%) | 14 higher on EKD |
| PP | T0 | 54.2 ± 40.7 | 75.4 ± 70.9 | Mean 17.6% (median -17.1%) | 13 higher on MPD |
| PP | T4 | 111.6 ± 86.0 | 127.5 ± 68.7 | Mean -5.7% (median -15.8%) | 15 higher on MPD |
| **PYY** | T0 | 155.0 ± 45.1 | 131.0 ± 33.1 | Mean 23.3% (median 22.6%) | 16 higher on EKD |
| **PYY** | T4 | 168.0 ± 59.9 | 127.00 ± 31.8 | Mean 36.4% (median 30.2%) | 17 higher on EKD |
| Total amylin | T0 | 65.4 ± 85.3 | 47.6 ± 60.1 | Mean 154.4% (median 5.3%) | 12 higher on EKD |
| Total amylin | T4 | 74.1 ± 103.6 | 49.1 ± 64.9 | Mean 184.7% (median 1.8%) | 10 higher on EKD |

#### Supplementary table 6: Meal consumption per dog, per visit at veterinary clinic prior to post-prandial blood glucose measurements, visual estimate of percentage of meal eaten. * denotes no data due to participant withdrawal.

| *Participant ID* | *BL meal consumption (%)* | *EKD meal consumption (%)* | *MPD meal consumption (%)* |
| --- | --- | --- | --- |
| 1 | 100 | 100 | 100 |
| 4 | 100 | 100 | 100 |
| 5 | 100 | 100 | 50 |
| 6 | 100 | 100 | * |
| 7 | 100 | 100 | 100 |
| 11 | 100 | 100 | 100 |
| 12 | 50 | 33 | 25 |
| 16 | 100 | 100 | 100 |
| 18 | 100 | 100 | 100 |
| 21 | 100 | 100 | 100 |
| 22 | 100 | 100 | 100 |
| 25 | 66 | 75 | 75 |
| 27 | 75 | 50 | 75 |
| 28 | 75 | 75 | 50 |
| 31 | 100 | 66 | 75 |
| 32 | 100 | 100 | 100 |
| 33 | 100 | 100 | 100 |
| 34 | 100 | 100 | 100 |
| 35 | 100 | 100 | 50 |
| 38 | 25 | 25 | 50 |

#### Supplementary table 7. Summarised data for blood glucose responses with mixed effects analysis and Tukey’s multiple comparisons test. Values expressed as mean ± SD. Analyses include all dogs with available glucose measurements (n = 23); one dog withdrew during the study and contributed partial data only.

|  | *Baseline* | *EKD* | *MPD* | *Fixed effect (Diet) p-value* | *Pairwise comparisons* |
| --- | --- | --- | --- | --- | --- |
| AUC_total_ | 1395.4 ± 219.7 | 1423.4 ± 183.0 | 1288 ± 164.2 | 0.017 | EKD > MPD (*p =* 0.0056) |
| Peak conc., (mmol/L) | 6.7 ± 0.2 | 6.9 ± 0.3 | 6.3 ± 0.2 | 0.034 | EKD > MPD (*p =* 0.0076) |
| Time to peak (min) | 81 ± 16.4 | 102 ± 18.6 | 42.6 ± 13.5 | 0.026 | EKD > MPD (*p =* 0.0050) |


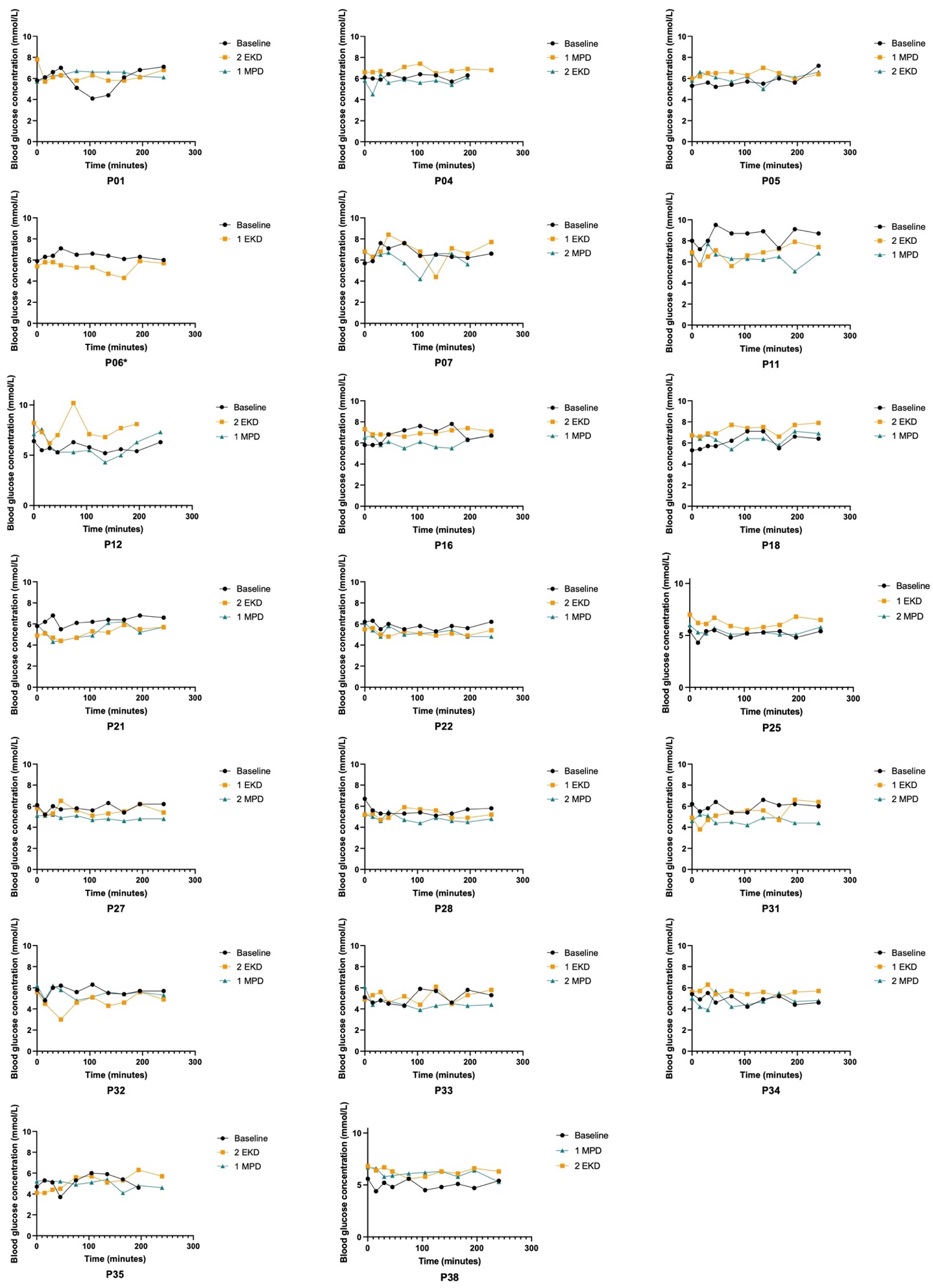


#### Supplementary figure 6: Blood glucose response curves per participant, by diet. Number in legend denotes sequence of diet (i.e. 1 = first, 2 = second).


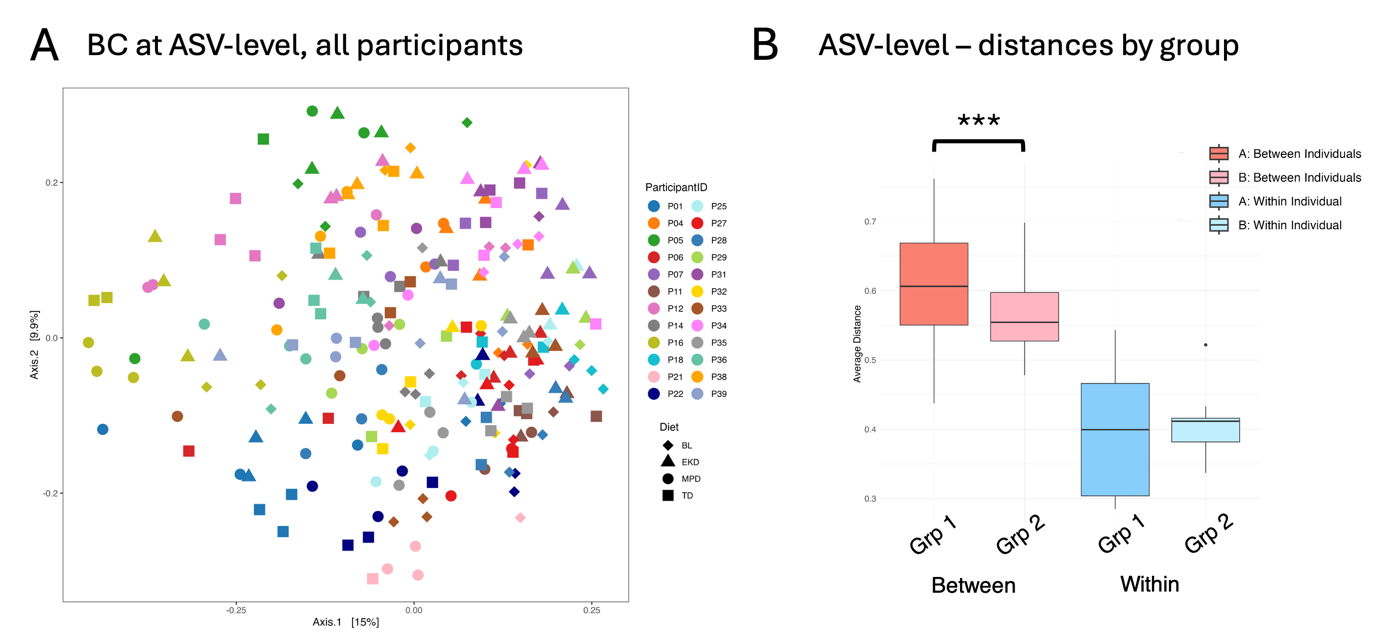


#### Supplementary figure 7: (A) PCoAs using ASV-level Bray-Curtis dissimilarity, for Group 1 and 2 treatment groups combined (n = 231 samples); (B) Average Bray–Curtis distances between samples from the same individual (within-individual) and between different individuals (between-individual), stratified by treatment group at ASV level. Differences were assessed using one-way ANOVA with Tukey’s post-hoc test (*** *p* < 0.001).

[submitted as separate PDF file to maintain resolution]

#### Supplementary figure 8: Heatmap of CLR-transformed relative abundance of ASVs. Rows are ordered according to the phyloseq phylogenetic tree, without additional clustering, and columns are ordered by Group, Diet, and Participant. ASVs identified as differentially abundant by LEfSe are indicated by coloured labels (orange = EKD-enriched; turquoise = MPD-enriched). Although these ASVs were significantly associated with diet at the group level, visual inspection demonstrates inter-individual variability in magnitude and direction of response. ASV identifiers correspond to sequence variants deposited in ENA.


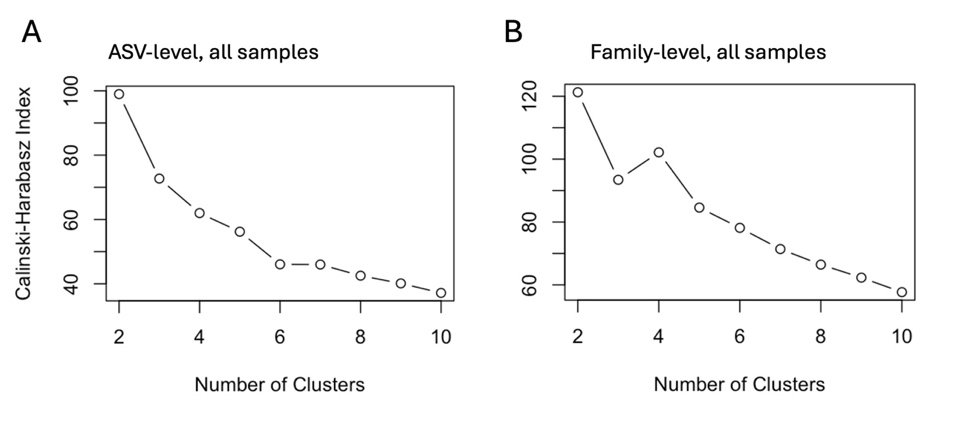


#### Supplementary figure 9: Calinski-Harabasz indices calculated for 2-10 clusters, using (A) CLR-transformed ASV-level abundance and (B) CLR-transformed Family-level abundance.


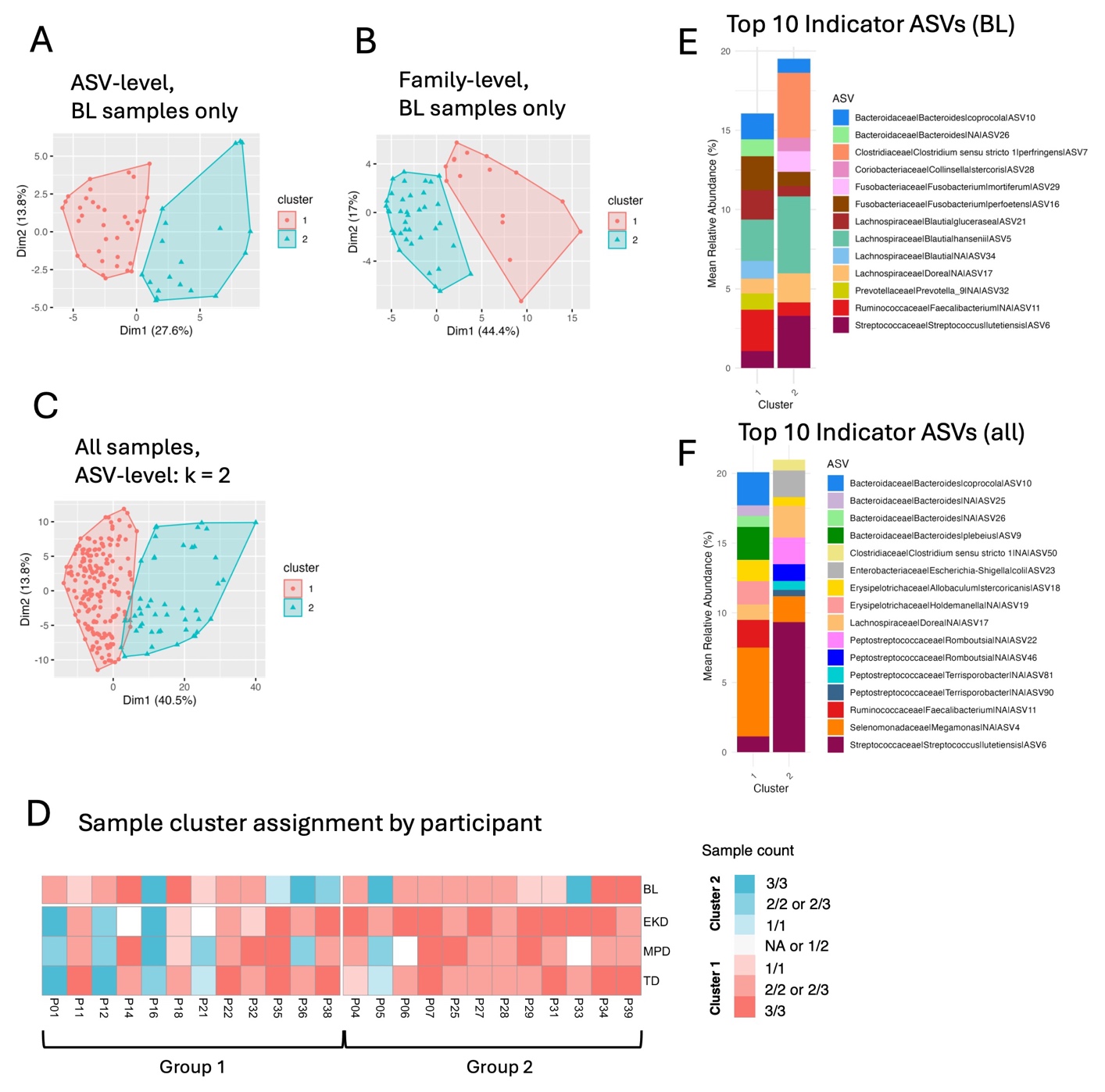


#### Supplementary figure 10: PAM clustering analysis using Bray Curtis dissimilarity. (A & B) ASV-level and family-level cluster assignment when k = 2 for BL samples only, n = 55; (C) ASV-level cluster assignment when k = 2 for all samples, n = 231; (D) Heatmap of k = 2 cluster assignment of samples by participant, separated by diet sequence (n = 0 – 3 samples per diet). Cell colour indicates sample count for that cluster where a cluster majority can be determined, i.e. if all samples or 2/3 samples are assigned to the same cluster. White colouring indicates if n = 0, or n = 2 and samples are assigned to both clusters. Relative abundance of top 10 indicator ASVs per cluster using multi-level pattern analysis, with all p < 0.05, for (F) BL samples only and (G) all samples combined.


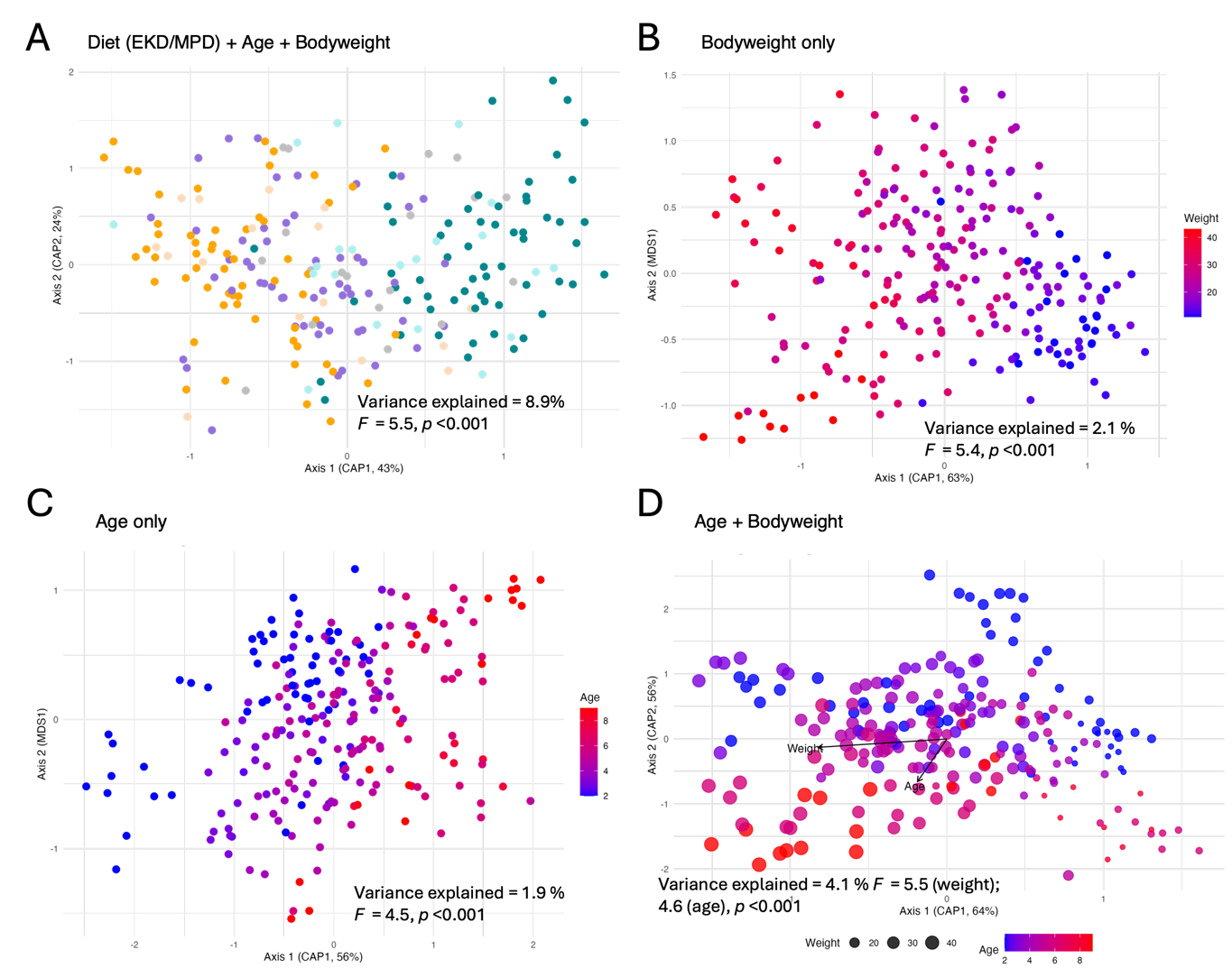


#### Supplementary figure 11: Distance-based redundancy analysis (db-RDA) using capscale on ASV-level Bray–Curtis dissimilarity (n = 231 samples). Models were fitted using: (A) diet (proportions), age, and bodyweight as explanatory variables; (B) bodyweight (dog size) as a single explanatory variable; (C) age as a single explanatory variable; and (D) age and bodyweight combined as explanatory variables. Statistical significance of each model was assessed using permutation ANOVA (999 permutations).


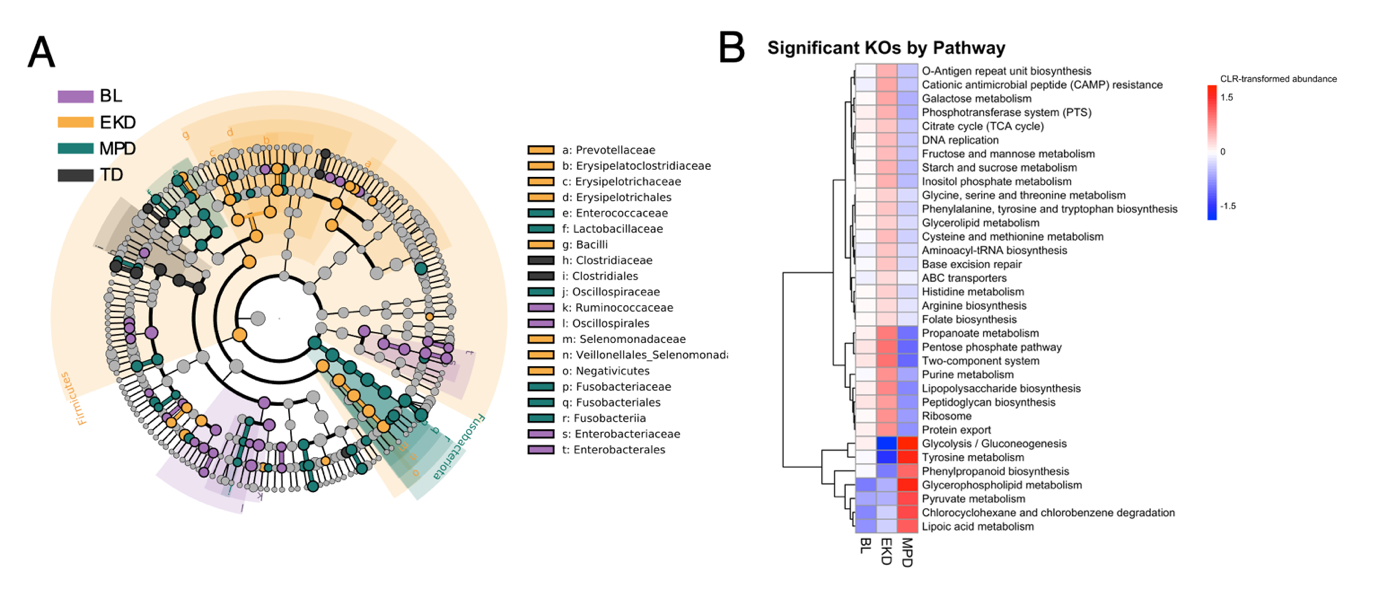
Supplementary figure 12: (A) Cladogram generated using LEfSe, showing taxa from phylum (outer ring) to species (inner ring). Each circle represents a taxon; taxa significantly enriched in a given diet are highlighted by diet colour, with larger circles indicating stronger associations based on LDA score (LDA > 2, p < 0.05). (B) Heatmap of CLR-transformed abundance of significantly enriched KEGG Orthologs (KOs), grouped by pathway. KOs were identified using LEfSe and pairwise Wilcoxon tests comparing EKD and MPD (LDA > 1, p < 0.05). Baseline (BL) samples are shown for reference.

####
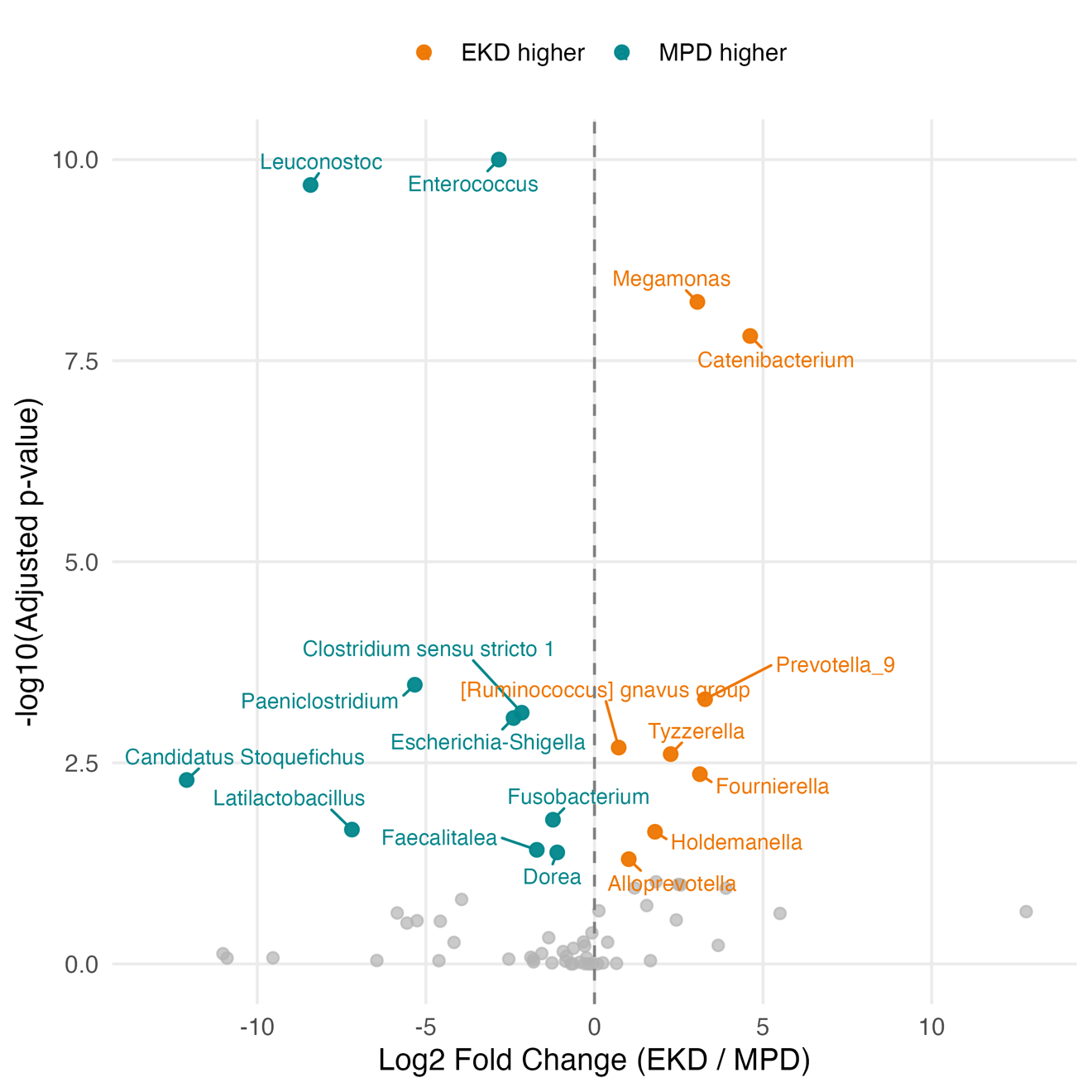


#### Supplementary figure 13. Volcano plot of genus-level differential abundance identified using ALDEx2 on CLR-transformed data. The x-axis shows log2 ratios calculated from the mean CLR-transformed abundance on EKD relative to MPD (log2[EKD/MPD]), such that positive values indicate enrichment on EKD and negative values indicate enrichment on MPD. Coloured points (orange = EKD-associated; turquoise = MPD-associated) denote genera with Benjamini-Hochberg FDR-corrected p-values < 0.05, as determined by statistical testing implemented in ALDEx2.


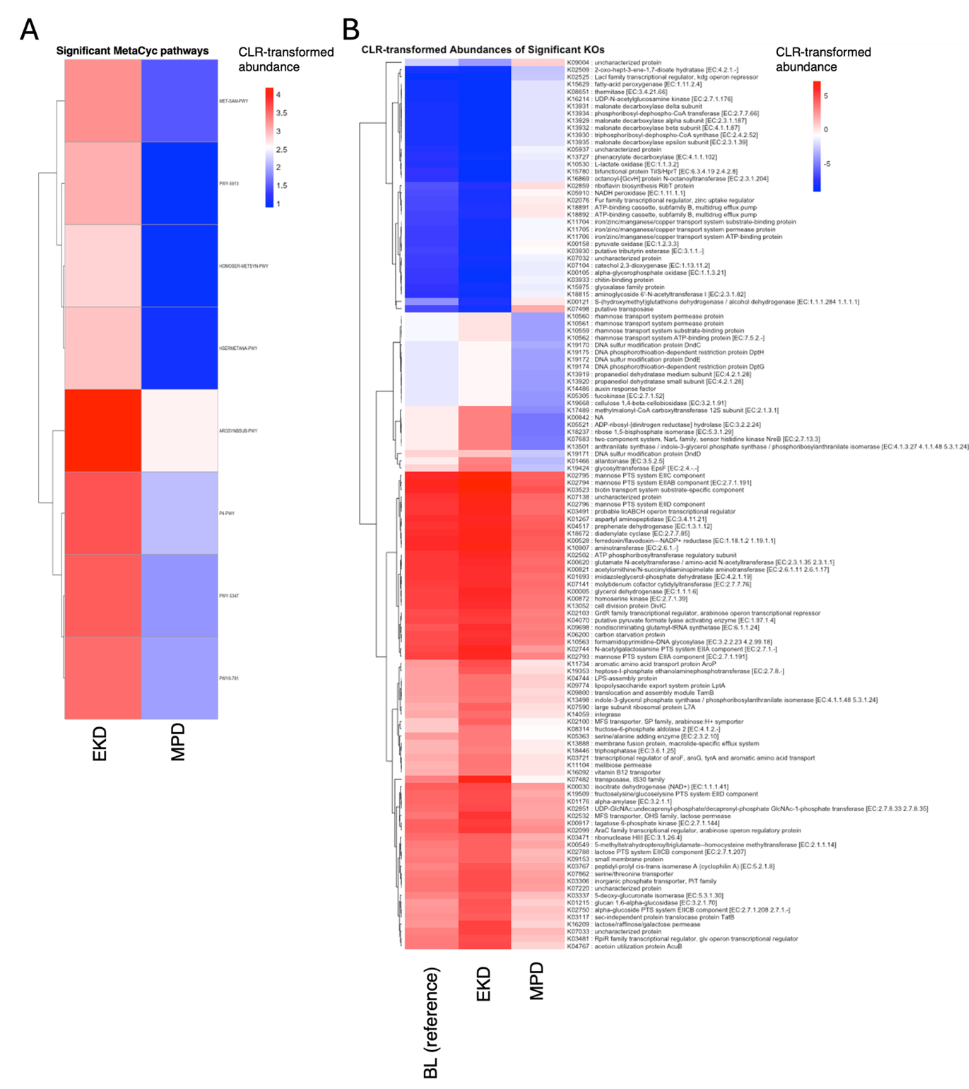


*Supplementary figure 14: (A) CLR-transformed relative abundance of MetaCyc pathways inferred by PICRUSt2 and identified as significantly differentially abundant between EKD and MPD using ALDEx2. (B) CLR-transformed relative abundance of KEGG Orthologs (KOs) inferred by PICRUSt2 and identified as significantly differentially abundant between EKD and MPD using ALDEx2. Heatmap values represent CLR-transformed abundances averaged across dogs for BL, EKD and MPD and are shown for visual comparison.*
