# Supplementary material for: Diet-induced metabolic and faecal microbiome responses in pet dogs fed a minimally processed versus extruded kibble diet
Source: Front Vet Sci. 2026 Apr 10;13:1734572. doi: 10.3389/fvets.2026.1734572 (PMC13107655; doi:10.3389/fvets.2026.1734572)
Supplement: Supplementary file 1 [file Supplementary Image 8.PDF › Supplementary Material/Supplementary Image 8.PDF]

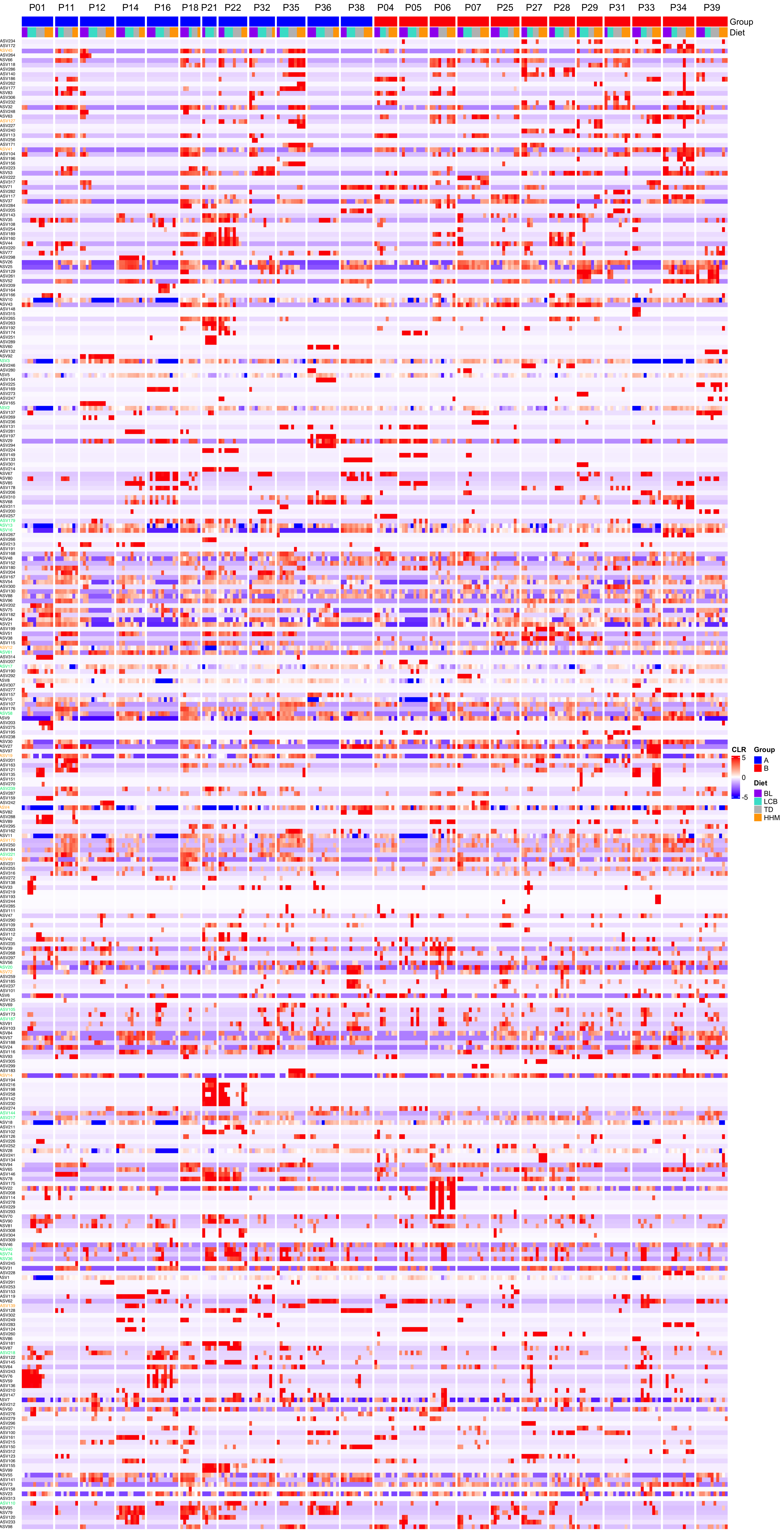
P01
P11
P12
P14
P16
P18
P21
P22
P32
P35
P36
P38
P04
P05
P06
P07
P25
P27
P28
P29
P31
P33
P34
P39
Group
Diet
CLR
5
0
-5
Group
A
B
Diet
BL
LCB
TD
HHM
